# The diffusible signal factor synthase, RpfF, in *Xanthomonas oryzae* pv. *oryzae* is required for the maintenance of membrane integrity and virulence

**DOI:** 10.1111/mpp.13148

**Published:** 2021-10-26

**Authors:** Prashantee Singh, Raj Kumar Verma, Subhadeep Chatterjee

**Affiliations:** ^1^ Laboratory of Plant Microbe Interactions Centre for DNA Fingerprinting and Diagnostics Uppal India; ^2^ Graduate Studies Manipal Academy of Higher Education Mangaluru India

**Keywords:** fatty acid synthesis, lipid A, membrane integrity, phospholipid, virulence

## Abstract

The *Xanthomonas* group of phytopathogens communicate with a fatty acid‐like cell–cell signalling molecule, *cis*‐11‐2‐methyl‐dodecenoic acid, also known as diffusible signal factor (DSF). In the pathogen of rice, *Xanthomonas oryzae* pv. *oryzae*, DSF is involved in the regulation of several virulence‐associated functions, including production and secretion of several cell wall hydrolysing type II secretion effectors. To understand the role of DSF in the secretion of type II effectors, we characterized DSF synthase‐deficient (*rpfF*) and DSF‐deficient, type II secretion (*xpsE*) double mutants. Mutant analysis by expression analysis, secretion assay, fatty acid analysis, and physiological studies indicated that *rpfF* mutants exhibit hypersecretion of several type II effectors due to a perturbed membrane and DSF is required for maintaining membrane integrity. The *rpfF* mutants exhibited significantly higher uptake of 1‐*N*‐phenylnapthylamine and ethidium bromide, and up‐regulation of *r* *poE* (σ^E^). Increasing the osmolarity of the medium could rescue the hypersecretion phenotype of the *rpfF* mutant. The *rpfF* mutant exhibited highly reduced virulence. We report for the first time that in *X*. *oryzae* pv. *oryzae* RpfF is involved in the maintenance of membrane integrity by playing a regulatory role in the fatty acid synthesis pathway.

## Graphical abstract

We show that in *Xanthomonas* quorum sensing plays an important role in the regulation of envelope stress and maintenance of membrane integrity, which is crucial for virulence.

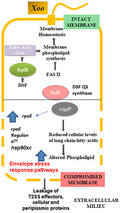

## 1 INTRODUCTION

Bacterial pathogens have to cope with and swiftly adapt to a wide range of stressful environmental conditions during their lifecycle, particularly during infection of a host (An et al., 2020). Bacterial stress adaptation often requires major metabolic reprogramming that involves coordinated changes in the cell transcriptome, proteome, and metabolome, along with cellular envelope remodelling (Needham & Trent, 2013; Rowlett et al., 2017). Envelope stress responses, including the ability to alter membrane phospholipids, O‐antigens, lipopolysaccharides, and fatty acid composition, are crucial for the maintenance of this barrier for bacterial survival and optimal virulence factor secretion (Albanesi et al., 2020; Hews et al., 2019).

In bacteria, cell population density sensing plays an important role in the regulation of several social behaviours such as secondary metabolite production, antibiotic resistance, lifestyle transition, virulence factor secretion, and response to environmental stresses (Bronesky et al., 2016; Chatterjee et al., 2020; Pena et al., 2019). To do this, bacteria use a cell–cell communication process called quorum sensing (QS), which integrates environmental cues, cell density, and cell history by coordinating multiple signalling pathways (LaSarre & Federle, 2013; Lee & Zhang, 2015; Moradali et al., 2017; Moreno‐Gàmez et al., 2017; Rutherford & Bassler, 2012). This enables bacteria to synchronize the expression of energetically expensive biological processes as a collective only when the impact of those processes on the environment or a host will be maximized (Pena et al., 2019). QS relies on the production, release, accumulation, and population‐wide detection of extracellular small diffusible signal molecules called autoinducers (Chatterjee et al., 2020; Eickhoff & Bassler, 2018).

The *Xanthomonas* group of phytopathogens communicates with a fatty acid‐like cell–cell signalling molecule, *cis*‐11‐2‐methyl‐dodecenoic acid, also known as the diffusible signal factor (DSF) (Barber et al., 1997; He et al., 2010; Zhou et al., 2015). Synthesis and perception of the DSF signal require products of the *rpf* (regulator of pathogenicity factor) gene cluster (*rpfF*, *rpfB*, *rpfC*, and *rpfG* that encode DSF synthase, fatty Acyl‐CoA ligase, sensor, and response regulator, respectively) (An et al., 2014; Andrade et al., 2006; Bi et al., 2014; Ryan et al., 2015; Slater et al., 2000; Zhou et al., 2017). In this study, we examine the role of DSF synthase (RpfF) in maintaining the membrane integrity and membrane function, as it is essential for bacterial fitness, as well as the effective establishment of infection in harsh and stressful environmental conditions both within and outside the host (Chatterjee & Sonti, 2002; Chatterjee et al., 2020; Hews et al., 2019; Rai et al., 2015).

Our previous study revealed that an *rpfF* mutant of *Xanthomonas oryzae* pv. *oryzae* (Xoo) exhibits atypical behaviour of DSF‐regulated traits, with hyper‐release of type II secretion system (T2SS) effectors, and additionally a deficiency in growth and virulence (Rai et al., 2012). However, the precise role of RpfF in hyper‐release and/or membrane damage is still elusive. Here, we show that the *rpfF* mutant of Xoo hyper‐releases the T2SS effectors independently of the type II secretion machinery and also shows leakage of periplasmic and cytoplasmic proteins. This finding suggests the compromised state of the cell envelope and employment of stress adaption response in the *rpfF* mutant. This study provides insight into the physiological role of RpfF in membrane homeostasis and adaptation to environmental stress, probably through its regulatory role in envelope homeostasis and stress response pathways.

## 2 RESULTS

### 2.1 The hyperextracellular release of T2SS effectors in DSF synthase *rpfF* mutant of Xoo is independent of the T2SS

To understand whether the hyper‐release of the T2SS effectors was due to a compromised cell envelope or due to uncontrolled activity of the T2SS, we constructed a T2SS single mutant Δ*xpsE*/pHM1 (mutant of the ATPase component of T2SS, deficient in the extracellular secretion of T2SS effectors; pHM1 vector control), an *rpfF* T2SS double mutant Δ*xpsE*Δ*rpfF*/pHM1, and *rpfF* complemented strains Δ*rpfF*/pSC9 (pSC9:*rpfF* containing complementing plasmid in pHM1) and Δ*xpsE*Δ*rpfF*/pSC9, and analysed the secretome of these strains.

**FIGURE 1:**
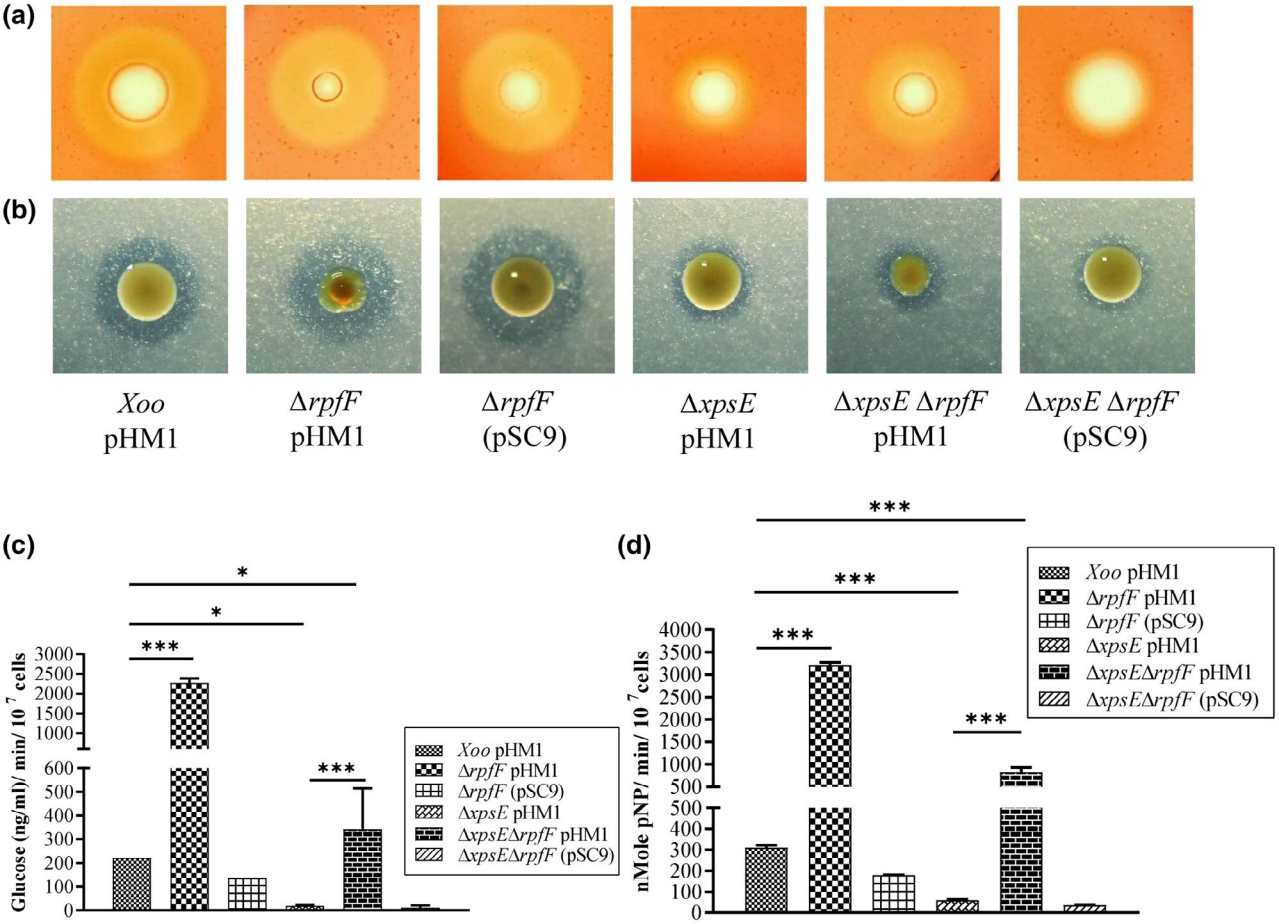
Diffusible signal factor synthase (*rpfF*)‐deficient mutants hypersecrete type II secretion system‐specific effectors. Cellulase and lipase secretion assays were performed in *Xanthomonas oryzae* pv. *oryzae* (Xoo) strains carrying either the empty vector pHM1 (as vector control) or the recombinant vector pSC9, which had the full‐length *rpfF* gene cloned into pHM1 (for complementation). (a) Xoo strains were spotted on peptone sucrose agar (PSA) plates supplemented with 0.2% carboxymethyl cellulose (CMC) and stained with Congo red to determine the extent of cellulase secretion. (b) Plate assay for lipase activity was carried out on PSA supplemented with 0.5% tributyrin in 100 mM Tris (pH 8) and 25 mM calcium chloride, and the ratio of halo to colony diameter determined the extent of lipase secretion. Extracellular protein fractions were isolated and assayed for (c) cellulase activity using 2% CMC as substrate and calculated as ng/ml of glucose released per minute per 10^7^ cells by the dinitrosalicylic acid method, and (d) lipase activity using *p*‐nitrophenyl butyrate as substrate and activity calculated as nmol of *p*‐nitrophenol (pNP) released per minute per 10^7^ cells. The experiments were performed in three biological replicates. Vertical error bars represent *SD*. ***p < 0.001, **p < 0.002, *p < 0.005 as determined by a one‐way analysis of variance followed by post hoc Tukey honestly significant difference (HSD) analysis

The β‐1,4‐endoglucanase (cellulase) and lipase plate assays indicated that the *rpfF* mutant had a significantly higher ratio of halo to colony diameter compared to the wild type and the complemented strain. The *rpfF* T2SS double mutant exhibited a ratio of a similar level to that of the wild type. This is in contrast to the phenotype exhibited by the single T2SS mutant and the complemented strain, which demonstrated a minimal ratio, indicative of a nonfunctional T2SS (Figure 1a,b).

The colourimetric assays for enzyme activity of cell number‐normalized extracellular protein extracts revealed that the *rpfF* mutant exhibited higher cellulase and lipase activity (10.5‐ and 10.4‐fold, respectively) compared to the wild type (Figure 1c,d). Additionally, the T2SS mutant showed reduced cellulase and lipase activity (10.2‐ and 5.2‐fold, respectively), whereas the T2SS *rpfF* double mutant exhibited higher cellulase and lipase activity (1.5‐ and 2.7‐fold, respectively) compared to the wild‐type strain (Figure 1c,d). This finding suggests the presence of an altered cell envelope in the *rpfF* mutant.

To support this notion, we performed sodium dodecyl sulphate‐polyacrylamide gel electrophoresis (SDS‐PAGE), silver staining, and western blotting probed with anticellulase and antilipase antibodies of cell number‐normalized extracellular protein fractions grown in peptone sucrose (PS) medium supplemented with and without 5% sucrose (osmoprotectant).

**FIGURE 2:**
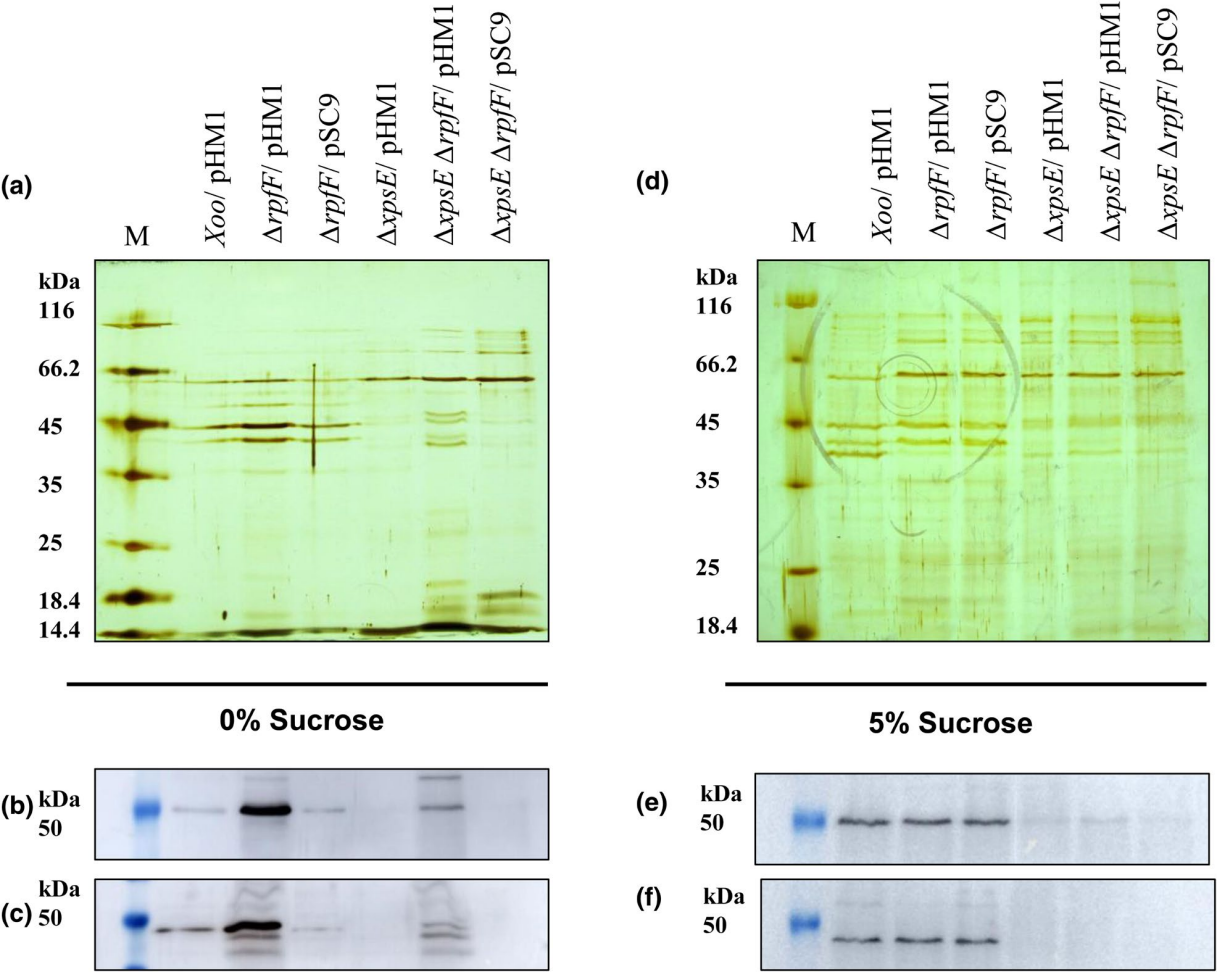
Hyper‐release of protein content by diffusible signal factor synthase (Δ*rpfF*) mutant and type II secretion system (Δ*xpsE*) Δ*rpfF* double mutant is restored to wild‐type level by 5% sucrose supplementation. (a) Silver‐stained SDS‐PAGE profiles of cell number‐normalized extracellular protein fractions of the *Xanthomonas oryzae* pv. *oryzae* (Xoo) strains. Western blots of the corresponding extracellular fractions of the Xoo strains probed with (b) anticellulase and (c) antilipase antibodies. (d) Silver‐stained SDS‐PAGE profile of the cell number‐normalized extracellular protein fractions of the Xoo strains grown in peptone sucrose (PS) medium supplemented with 5% sucrose. Western blot of the corresponding extracellular fractions of the Xoo strains grown in PS supplemented with 5% sucrose probed with (e) anticellulase and (f) antilipase antibodies

In the absence of sucrose, the silver‐stained SDS‐PAGE profile and corresponding western blots probed with anticellulase and antilipase antibodies showed a higher level of extracellular protein, as well as a greater level of cellulase and lipase in the western blots for the *rpfF* mutant when compared to the wild type and the complemented strain (Figure 2a–c). Correspondingly, the T2SS *rpfF* (Aparna et al., 2009) double mutant showed higher protein as well as cellulase and lipase levels compared to the T2SS single mutant and the double mutant complemented with the full‐length *rpfF* gene (Figure 2a–c).

As expected, the leaky outer membrane phenotype could be corrected with supplementation of osmoprotectant (5% sucrose added to the growth medium): the extracellular protein levels of the *rpfF* and T2SS *rpfF* double mutants returned back to that of the wild type and T2SS single mutant, respectively (Figure 2d). This was further confirmed by western blots that displayed an equal level of cellulase and lipase in the culture supernatants of the wild type and the *rpfF* mutant strains, and an almost nil level of cellulase and lipase in the T2SS mutants (Figure 2e,f).

### 2.2 Deletion of *rpfF* causes leakage of non‐type II secreted proteins due to leaky membrane

**FIGURE 3:**
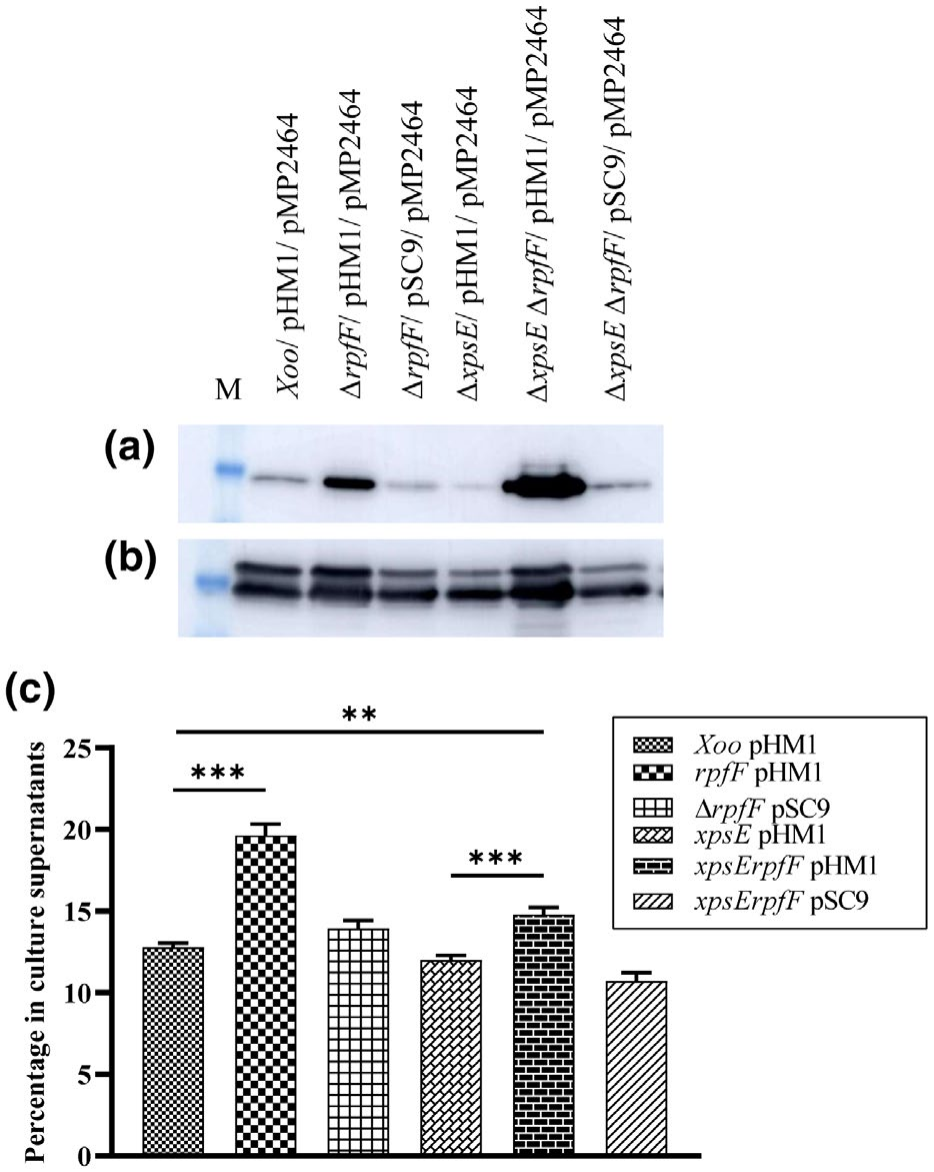
Lack of diffusible signal factor synthase (*rpfF*) causes leakage of intracellular proteins into the extracellular milieu. (a) Extracellular and (b) whole‐cell protein fractions of the *Xanthomonas oryzae* pv. *oryzae* (Xoo) strains Xoo/pHM1/pMP2464, Δ*rpfF*/pHM1/pMP2464, Δ*rpfF*/pSC9/pMP2464, Δ*xpsE*/pHM1/pMP2464, Δ*xpsE*Δ*rpfF*/pHM1/pMP2464, and Δ*xpsE*Δ*rpfF*/pSC9/pMP2464 were probed with anti‐enhanced green fluorescent protein (EGFP ) antibody. pHM1 is the cosmid expression vector, pSC9 is pHM1 with the full‐length *rpfF* gene, and pMP2464 is the recombinant plasmid expressing EGFP. (c) Alkaline phosphatase activity of the cell number normalized‐extracellular and whole‐cell protein fractions of the Xoo strains Xoo/pHM1, Δ*rpfF*/pHM1, Δ*rpfF*/pSC9, Δ*xpsE*/pHM1, Δ*xpsE*Δ*rpfF*/pHM1, and Δ*xpsE*Δ*rpfF*/pSC9 was determined using *p*‐nitrophenol phosphate as the substrate and the percentage of extracellular alkaline phosphatase in the extracellular protein fractions was then plotted. All experiments were performed as three biological triplicates. Vertical error bars represent *SD*. ***p < 0.001, **p < 0.002 as determined by a one‐way analysis of variance followed by post hoc Tukey honestly significant difference (HSD) analysis

To examine whether the leakage of proteins was restricted to the T2SS effectors and whether the cytoplasmic and periplasmic proteins were also being leaked into the extracellular milieu, we performed western blotting probed with anti‐enhanced green fluorescent protein (EGFP) antibody using the extracellular protein fractions of the cell number‐normalized Xoo strains harbouring the plasmid pMP2464, a derivative of pBBR1MCS5 containing a constitutively expressed *egfp* gene (Pradhan et al., 2012; Stuurman et al., 2000). The secretome of the *rpfF* mutant Δ*rpfF*/pHM1/pMP2464, as well as the T2SS and *rpfF* double mutant Δ*xpsE*Δ*rpfF*/pHM1/pMP2464, displayed a significantly higher level of EGFP (Figure 3a). However, the whole‐cell protein fractions of all the strains exhibited an approximately equal level of EGFP (Figure 3b).

Additionally, a spectrophotometric assay to measure the percentage of alkaline phosphatase activity in the extracytoplasmic protein fractions was performed using *p*‐nitrophenyl phosphate as the substrate (Sikora et al., 2007). The *rpfF* mutant and the T2SS *rpfF* double mutant in comparison to the wild‐type strain displayed a significantly higher percentage of alkaline phosphatase activity in the extracellular milieu relative to the whole‐cell protein fractions (Figure 3c).

### 2.3 Xoo *rpfF*‐deficient mutant is more permeable to ethidium bromide and 1‐*N*‐phenylnapthylamine, indicative of compromised membrane permeability

The permeability of Xoo strains was assessed by measuring the uptake of the fluorescent dyes ethidium bromide (EtBr) (fluoresces only after permeating both outer and inner membrane, and intercalating into nucleic acid) and 1‐*N*‐phenylnapthylamine (NPN; a nonpolar dye that fluoresces strongly in hydrophobic environments, such as the lipid bilayer of the inner membrane or the inner leaflet of the outer membrane) (Zou et al., 2012).

To study the uptake kinetics of both EtBr and NPN and to minimize the extrusion of these compounds by the AcrAB‐TolC system, the effect of *rpfF* mutation was seen in a *tolC* mutant background. A *tolC* mutant Δ*tolC*/pHM1, double mutant Δ*tolC*Δ*rpfF*/pHM1, and *tolC rpfF* double mutant complemented with the full‐length *rpfF* gene Δ*tolC*Δ*rpfF*/pSC9 were constructed for EtBr and NPN uptake assays. The percentage fluorescence observed revealed that the *rpfF tolC* double mutant Δ*tolC*Δ*rpfF*/pHM1 showed approximately 3–4‐fold higher uptake of both NPN and EtBr compared to the single mutant Δ*tolC*/pHM1 as well as the double mutant complemented with the full‐length *rpfF* gene Δ*tolC*Δ*rpfF*/pSC9 (Figure S1a,b).

### 2.4 Inactivation of *rpfF* leads to sensitivity to detergents, which causes cell envelope stress

The striking consequence of *rpfF* deletion on the Xoo cell envelope led us to investigate the response of strains with compromised membranes to environmental stress (Rowlett et al., 2017; Zou et al., 2012). Mutants defective in membrane integrity exhibit increased sensitivity to detergents and to antimicrobial compounds (Kingston et al., 2011). To access the stress response, we measured the effects of various detergents (Tween 20, Triton X‐100, and SDS) on the growth of Δ*rpfF*/pHM1. The mutant exhibited higher sensitivity to these detergents compared to the wild type or the complemented strain (*rpfF* mutant harbouring the wild‐type allele) (Figure S2).

### 2.5 Xoo *rpfF* is required for in planta migration and virulence

Chatterjee and Sonti (2002) reported that the *rpfF* mutant of Xoo is virulence‐deficient compared to the wild type. To investigate the underlying basis of this virulence deficiency, we performed in planta migration, survival, and virulence assays by the wound inoculation method (Verma et al., 2018).

**FIGURE 4:**
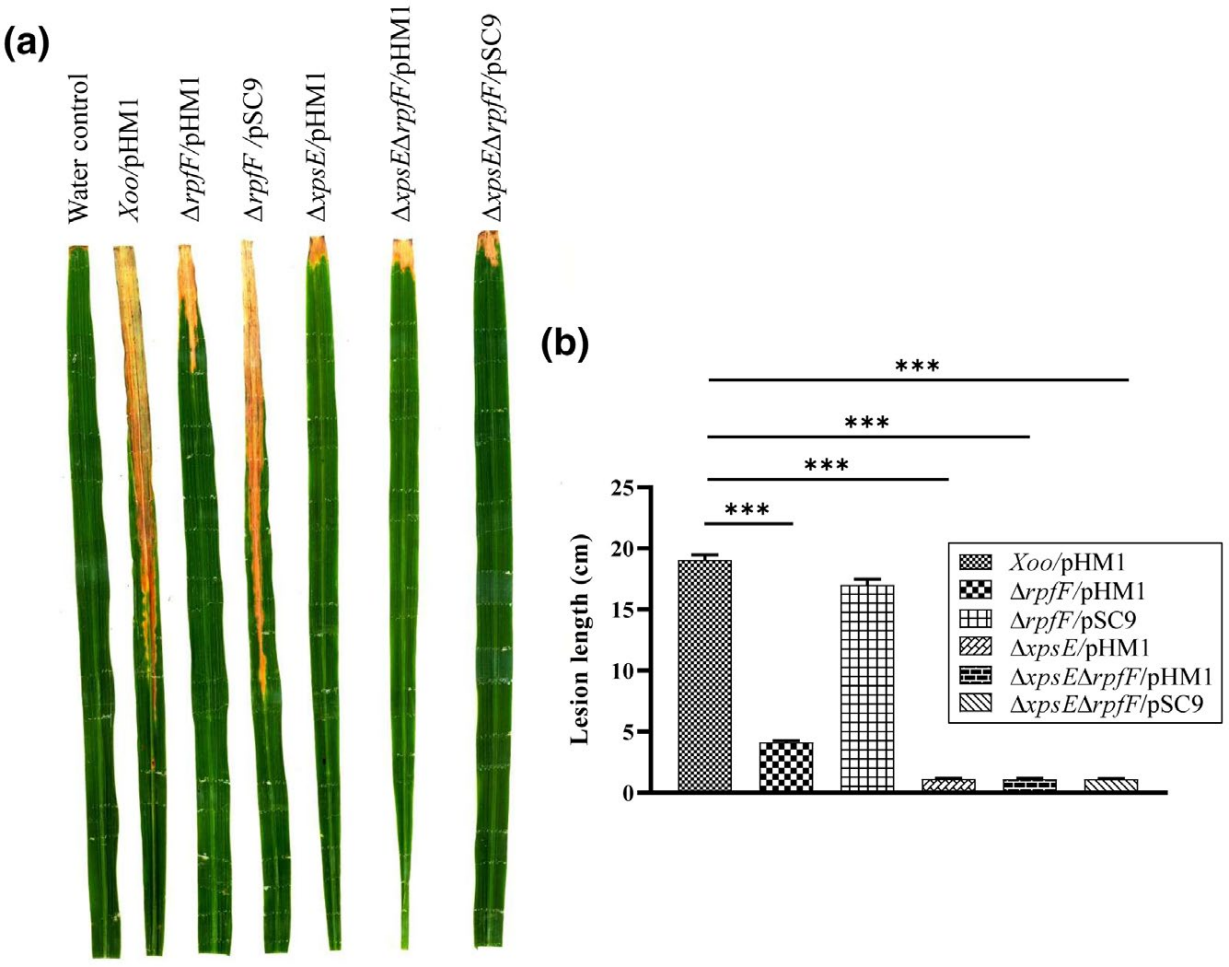
*rpfF* deficiency causes a significant reduction in the virulence of *Xanthomonas oryzae* pv. *oryzae* (Xoo). Approximately 10^9^ cells were used to clip inoculate 40‐ to 45‐day‐old susceptible rice plants (cv. Taichung Native 1). The lesion length was then measured. (a) Representative image of rice leaves inoculated with different strains of Xoo and (b) Mean lesion length of 15 individual leaves 14 days postinfection. The experiments were performed in three biological replicates. Vertical error bars represent *SD*. ***p < 0.001 as determined by a one‐way analysis of variance followed by post hoc Tukey honestly significant difference (HSD) analysis, calculated for the 14th day postinfection

**FIGURE 5:**
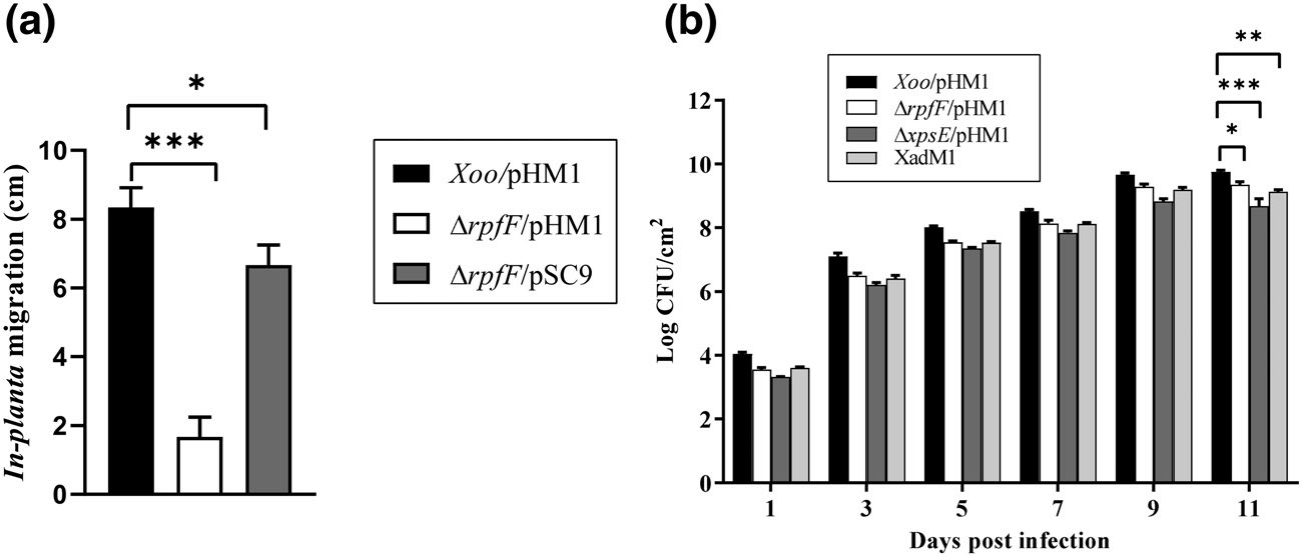
The *rpfF* mutant exhibits significantly reduced in planta migration and reduced survival. (a) In planta bacterial migration assay was performed 5 days postinoculation on leaves inoculated by the leaf clip method as described in section 4. Migration was estimated after 1–3 days by observing the bacterial ooze from the cut ends of the rice leaf pieces. The experiments were repeated three times. Vertical error bars represent *SD*. ***p < 0.0001, *p < 0.05 as determined by a one‐way analysis of variance followed by post hoc Tukey honestly significant difference (HSD) analysis. Both the experiments were repeated three times. (b) In planta colony‐forming units (cfu) measurements for different strains of *Xanthomonas oryzae* pv. *oryzae* (Xoo). The Xoo/pHM1, ∆*rpfF*/pHM1, ∆*xpsE*/pHM1, and XadM1 (adhesion mutant control) were clip inoculated into 40‐ to 45‐day‐old susceptible rice plants and cfu was determined from approximately 1 cm^2^ of the leaf by the serial dilution method as described in Section 4. Vertical error bars represent *SD*. ***p < 0.001, **p < 0.002, *p < 0.05 as determined by a one‐way analysis of variance followed by post hoc Tukey HSD analysis

The T2SS mutants Δ*xpsE*/pHM1, Δ*xpsE*Δ*rpfF*/pHM1, and Δ*xpsE*Δ*rpfF*/pSC9 exhibited significantly reduced lesion lengths compared to the wild‐type strain. Moreover, the *rpfF* mutant also demonstrated significantly reduced lesion lengths compared to the wild type but not as reduced as the T2SS mutant (Figure 4). The *rpfF* mutant exhibited significantly reduced migration, as we could not detect *rpfF* bacterial cells beyond 2 cm from the point of inoculation (Figure 5a). Because the *rpfF* mutant did not exhibit migration beyond 2 cm from the point of inoculation, we therefore repeated the in planta survival assay by measuring bacterial colony‐forming units (cfu) from the leaf region near the point of inoculation (within 1 cm leaf length from the point of inoculation). As a control, we also included an *XadM* (adhesion) mutant strain that had been earlier shown to be deficient in attachment and exhibited partial virulence deficiency (Pradhan et al., 2012). The *rpfF* mutant exhibited approximately 2.5‐fold lower in planta cfu on the 11th day after bacterial inoculation at the site of infection (Figure 5b).

These data suggested that the in planta migration and virulence of the *rpfF* mutant was more affected than its survival within the host (within the site of inoculation).

### 2.6 Lack of *rpfF* causes alteration in the lipopolysaccharide, phospholipid, and fatty acid profile of the Xoo envelope

Cell integrity and cell function are determined by the composition and physiological roles played by the building blocks of the cell envelope (Albanesi et al., 2020; Hews et al., 2019; Rowlett et al., 2017; Shibuya, 1992; Silhavy et al., 2006; Steeghs et al., 2001; Thomanek et al., 2019; Zhang & Rock, 2008). To correlate the membrane damage, increased susceptibility to environmental stress, and reduced in planta migration and survival seen in the *rpfF* mutant with alterations in cell envelope composition, we performed lipopolysaccharide (LPS) visualization by SDS‐PAGE, phospholipid analysis by thin‐layer chromatography (TLC), and fatty acid analysis by gas chromatography/mass spectrometry (GC/MS).

**FIGURE 6:**
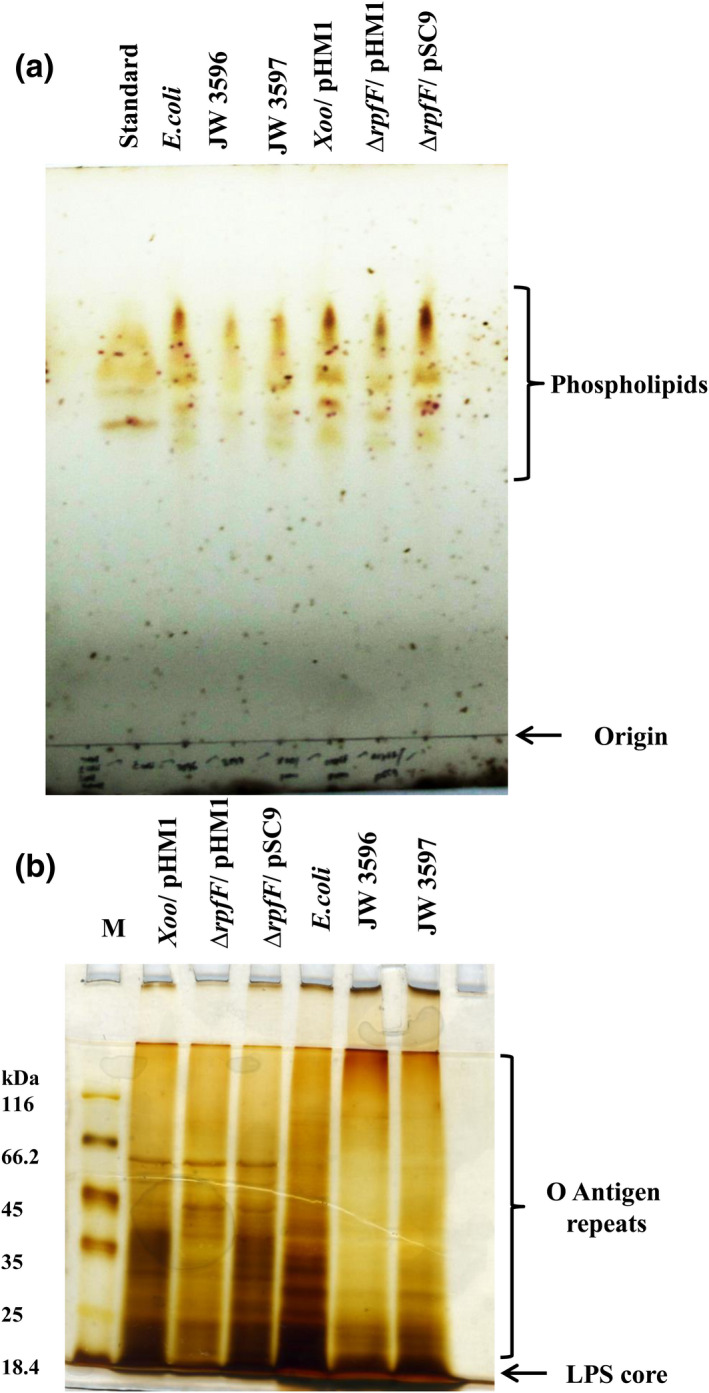
*rpfF* mutant shows an altered phospholipid and lipopolysaccharide (LPS) profile. (a) Thin‐layer chromatography analysis of the phospholipids extracted from sodium acetate‐treated optical density‐normalized cultures of the *Escherichia coli* wild‐type strain, and the *E. coli* LPS mutants JW3596 and JW3597 along with the *Xanthomonas oryzae* pv. *oryzae* (Xoo) strains Xoo/pHM1, ∆*rpfF*/pHM1, and ∆*rpfF*/pSC9. *E*. *coli* total lipid extract (Sigma) was used as standard. (b) LPS from the *E*. *coli* wild‐type strain and LPS mutants JW3596 and JW3597 along with the Xoo strains Xoo/pHM1, ∆*rpfF*/pHM1, and ∆*rpfF*/pSC9 were separated by SDS‐PAGE and visualized by silver staining. These experiments were performed as three biological replicates

The Xoo strains Xoo/pHM1, Δ*rpfF*/pHM1, and Δ*rpfF*/pSC9, and control strains *Escherichia coli* (wild type) and *E*. *coli* LPS mutants JW3596 (*rfaC*) and JW3597 (*rfaL*) were used for this study (Baba et al., 2006; Klena et al., 1992a, 1992b; Pagnout et al., 2019). As expected, the phospholipid profiles on TLC of the LPS mutants of *E*. *coli* and the *rpfF* mutant of Xoo were significantly altered compared to their respective wild‐type strains (Figure 6a). The silver‐stained LPS profile of the *rpfF* mutant was similar to that of the LPS mutants wherein the intensity of the O‐antigen repeats was significantly reduced (Figure 6b).

**FIGURE 7:**
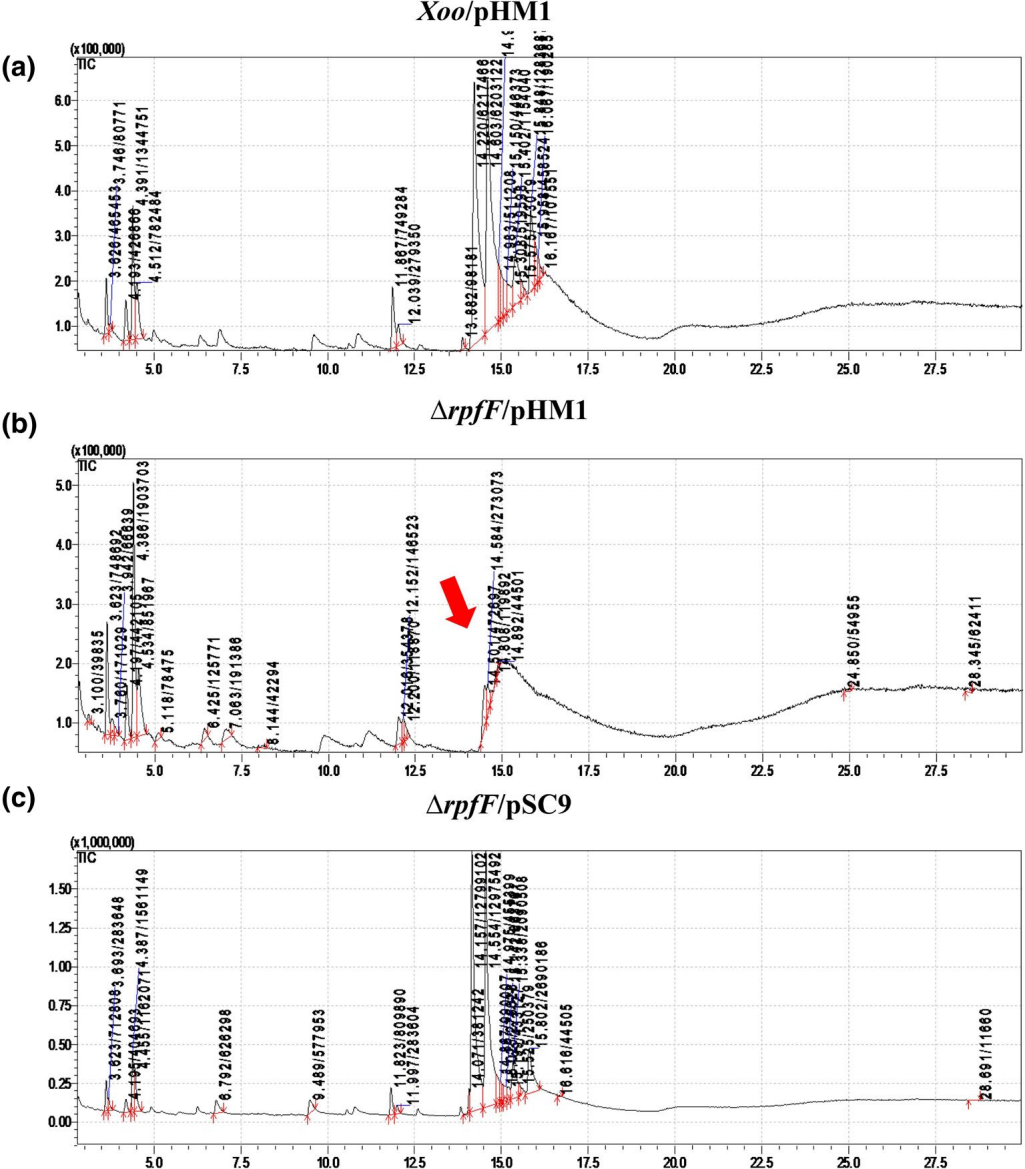
*rpfF* deficiency causes a change in the fatty acid profile of *Xanthomonas oryzae* pv. *oryzae* (Xoo). The total ion chromatogram of extracted fatty acid methyl esters of the Xoo strains was obtained by gas chromatography/mass spectrometry (GC/MS) analysis. Fatty acid methyl esters were extracted from 40 mg of freshly streaked Xoo strains Xoo/pHM1, ∆*rpfF*/pHM1, and ∆*rpfF*/pSC9 by a modified Bligh and Dyer method. The fatty acid methyl esters were then subjected to GC/MS analysis on a GC‐2010 Plus system (Shimadzu)

With fatty acids being the main components of phospholipids and fatty acid synthase (FAS) II intermediate β‐hydroxyacyl‐ACP being the precursor molecule of DSF, the total cellular fatty acid methyl esters from the Xoo strains were analysed by gas chromatography/mass spectrometry (GC/MS). The fatty acid profile of the *rpfF* mutant was distinct from that of the wild type and the complementary strain (Figure 7). The short‐chain fatty acids 7:0 and 5:0 that eluted with a retention time of 3.6–4.4 min were more prevalent in the *rpfF* mutant than the wild‐type and the complementary strains. However, the long‐chain fatty acids 9:0, 10:0, and 11:0 that were eluted with a retention time of 14.61–15.85 min were more prevalent in the wild‐type and complementary strains but they were either not detected or present in minimal amounts in the *rpfF* mutant (Table S1).

### 2.7 *rpfF* deletion‐induced membrane damage leads to the activation of stress response pathways

As activation of the σ^E^ stress response is an indication of outer membrane damage (Alba & Gross, 2004; Albanesi et al., 2020; Hews et al., 2019; Rouviere et al., 1995; Rowlett et al., 2017; Ruiz & Silhavy, 2005), we reasoned that increased expression of *rpoE* and genes involved in other well‐known stress response pathways in the *rpfF* mutant would be another means to ascertain the presence of envelope damage.

**FIGURE 8:**
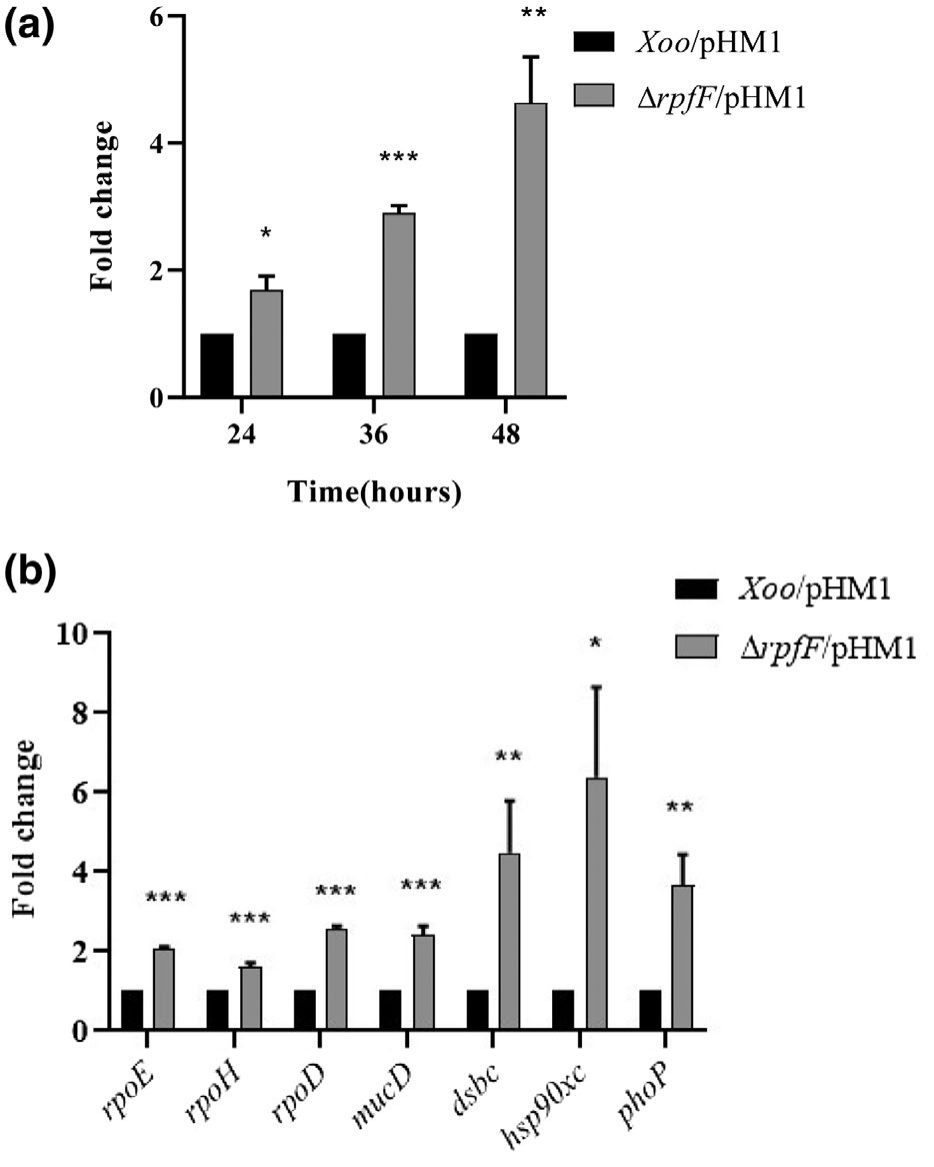
Envelope stress genes exhibit elevated expression in the *Xanthomonas oryzae* pv. *oryzae* (Xoo) *rpfF* mutant, signifying the presence of a compromised membrane. (a) Phase‐dependent expression analysis by reverse transcription quantitative PCR (RT‐qPCR) of the *rpoE* gene (encoding σ^E^). (b) RT‐qPCR of the genes *rpoE*, *rpoH*, *rpoD*, *mucD*, *dsbc*, *hsp90xc*, and *phoP*, which are involved in envelope stress response pathways. Relative quantification of the stress response genes by RT‐qPCR. RNA was isolated from the strains by the TRIzol method. The analysis was done by relative quantification software (Applied Biosystems) and the output data were analysed by fold change calculation (2^−ΔΔ^ *^C^* ^t^) with respect to the wild‐type strain using 16S rRNA as the endogenous control. Standard errors were calculated based on three independent experiments. The paired Student's *t* test was done to determine the significance of differences of the test strain compared to the wild‐type Xoo. *p ≤ 0.05, **p ≤ 0.01, ***p ≤ 0.001, error bars represent *SD*

Our phase‐dependent expression analysis by reverse transcription quantitative PCR (RT‐qPCR) of the *rpoE* gene revealed that *rpoE* was significantly up‐regulated in the Xoo *rpfF* mutant. The up‐regulation was approximately 2‐, 3‐, and 4‐fold in the log, late log, and stationary growth phases, respectively (Figure 8a). This indicates that as the growth of the *rpfF* mutant progresses from the log phase to the stationary phase, the stress on the envelope increases significantly. Other stress‐related genes and stress factors *rpoH* (σ^32^), *mucD*, *dsbC*, *hsp90xc*, and *phoP* (Rowlett et al., 2017) were also seen to be significantly up‐regulated in the *rpfF* mutant in the log phase cells (Figure 8b), further confirming the presence of a perturbed membrane and altered adaptability to stress response in the *rpfF* mutant.

## 3 DISCUSSION

The DSF regulatory system has been extensively studied over the last few decades in many xanthomonads and has been implicated in the metabolically costly production of virulence factors, including cell‐associated or secreted molecules, which is a survival strategy for pathogens to evade various environmental stresses and ensure effective disease progression (An et al., 2020; Büttner & Bonas, 2010; Chatterjee & Sonti, 2002; Chatterjee et al., 2020; Deng et al., 2016; Guo et al., 2012; He et al., 2010; Kakkar et al., 2015; Rai et al., 2012, 2015; Ryan et al., 2015).

Although Rai et al. (2012) reported the hypersecretion of T2SS effectors in the DSF synthase *rpfF* mutant of Xoo, little was known about its underlying mechanism. Here, we demonstrate that this atypical behaviour of the *rpfF* mutant is due to cell envelope damage and not due to the uncontrolled activity of the T2SS. We provide evidence that the T2SS *rpfF* double mutant secreted significantly higher levels of the T2SS effectors despite the absence of functional T2SS machinery (Figure 1a,b). In addition, the 5% sucrose supplementation in the growth medium (which might have osmotically protected the perturbed cell membrane from leakage) significantly suppressed overproduction of the T2SS effectors in the extracellular milieu (Figure 2a,b).

Leakage of cytoplasmic proteins to the extracellular milieu, a greater influx of fluorescent compounds, and increased sensitivity to detergents are phenotypes shown by different bacteria signifying the presence of a compromised membrane (Choi & Lee, 2019; Kawasaki & Manabe, 2010; Suzuki et al., 1978; Zou et al., 2012). In this study, we have provided several lines of evidence demonstrating the presence of a damaged membrane in the *rpfF* mutant, such as significantly higher leakage of alkaline phosphatase as well as EGFP in the secretome of the *rpfF* mutant and the T2SS *rpfF* double mutant (Figure 3), greater uptake of NPN and EtBr by the *tolC* *rpfF* double mutant compared to the *tolC* single mutant and the complement (Figure S1), increased level of sensitivity to detergents shown by the *rpfF* mutant (Figure S2), and reduced in planta growth, migration, and virulence (Figures 4 and 5).

In gram‐negative pathogenic bacteria, adaptation to environmental stress, particularly during host–pathogen interaction to promote survival and/or evade the immune system, is often accompanied by cellular envelope remodeling (Konovalova et al., 2017; Needham & Trent, 2013). The remodelling can occur in various parts of the envelope, from the capsule (Olaitan et al., 2014) to lipid A remodelling (Needham & Trent, 2013), change in LPS decoration (Chen & Groisman, 2013; Wang et al., 2015), and the protein content of the outer and/or inner membrane (Sikora et al., 2007), as well as alteration in the phospholipid (Rowlett et al., 2017) and fatty acid composition (Kumariya et al., 2015). The altered LPS, phospholipid, and fatty acid profiles displayed in the *rpfF* mutant provide insight into the probable cause for the envelope stress seen in the *rpfF* mutant of Xoo.

Bacterial cell envelope homeostasis is governed by several extracellular response systems that are activated by distinct environmental signals (Hews et al., 2019; Ruiz & Silhavy, 2005). We demonstrate that the inactivation of *rpfF* stimulates the activation of various sigma factors involved in the cellular stress response (σ^E^, the extracytoplasmic stress sigma factor, and σ^H^, the heat‐shock sigma factor) as well as the up‐regulation of genes that encode proteins involved in folding or degradation of polypeptides in the periplasm (Figure 8a,b), such as *mucD* (a homolog of the *E*. *coli* proteases *degP*/*htrA*), *hsp90xc* (a homolog of the *E*. *coli htpG*, regulated by σ^S^, the starvation/stationary phase sigma factor and implicated with binding unfolded proteins), *phoP* (regulated by PhoP/PhoQ), and *dsbC* (a thiol:disulphide oxidoreductase) (Rowlett et al., 2017; Rowley et al., 2006). Previous studies have reported that σ^E^ is activated in response to envelope damage and misfolded outer membrane proteins and controls the expression of several periplasmic chaperones, proteases, and LPS biosynthesis and outer membrane proteins that ensure proper outer membrane assembly (Alba & Gross, 2004; Albanesi et al., 2020; Ruiz et al., 2006). In our study, the increased *rpoE* up‐regulation as the cells entered the stationary phase of growth further corroborated the fact that the *rpfF* mutant experienced envelope stress (Figure 8a). These findings suggest that the inactivation of *rpfF* stimulates the activation of compensatory pathways, leading to different levels of cell envelope adaptability and cell function.

Previous studies have reported that the patterns of regulation of many of the homologous genes regulated by *rpfF* in the different species of *Xanthomonas*, as well as its close relatives, are strikingly different (An & Tang, 2018; Guo et al., 2012; He et al., 2006; He & Zhang, 2008; Huang et al., 2013; Huedo et al., 2014, 2015; Li et al., 2019; Rai et al., 2012; Siciliano et al., 2006; Wang et al., 2012; Zhou et al., 2015). The most intriguing difference relevant to our study was that while *rpfF* mutation in *Xanthomonas campestris* pv. *campestris* (Xcc) causes a reduction in the secretion of T2SS effectors (He et al., 2006), the same mutation in Xoo causes hyper‐release of the effectors (Rai et al., 2012).

Further studies done in Xcc showed that elevated RpfF activity in the *rpfB rpfC* double mutant causes a leaky membrane due to uninhibited cleavage of acyl‐acyl carrier protein (ACP) intermediates and release of free fatty acid in the extracellular milieu (Bi et al., 2014). This unhindered RpfF activity is rescued by RpfB, which has fatty acyl‐CoA ligase (FCL) activity that sequesters the free fatty acids from the extracellular milieu and reroutes them back to the membrane phospholipid biosynthesis pathway (Bi et al., 2014). RpfB has also been shown to be involved in DSF signal turnover in *Xanthomonas* (Wang et al., 2016). Expression analysis by RT‐qPCR of the *rpfB* gene showed that it was significantly down‐regulated in the *rpfF* mutant (Figure S3). It is possible that the *rpfF* mutant of Xoo has reduced FCL activity, which could be a plausible explanation for the altered phospholipid and fatty acid profile seen in the mutant (Figure S4). It is pertinent to note that it has been reported that the *rpfF* mutant of Xcc also exhibits an altered expression of several genes involved in fatty acid metabolism and fatty acid desaturase (He et al., 2006). Thus it appears that RpfF activity (Bi et al., 2012, 2014) needs to be optimal for the proper maintenance of membrane stability in both Xcc and Xoo. It is also possible that the change in membrane lipid composition or altered membrane could cause the pleotrophic effects exhibited by the *rpfF* mutant, of what appears to be DSF‐regulated traits (Dow et al., 2003; Dow, 2008).

In a previous study done on one of the *rpf* cluster variants of *Strephonomonas maltophilia*, a close relative of *Xanthomonas*, an elevated level of *iso*‐15:0 fatty acid was observed in the total cellular fatty acids. DSF and *iso*‐15:0 happen to share the common precursor molecule β‐hydroxyacyl‐ACP and the addition of *iso*‐15:0 to the culture medium substantially increased DSF production by the strain, thereby leading to speculation of a potential connection between DSF production and membrane synthesis (Heudo et al., 2015). Previous studies have also reported that altering the expression, blocking, or mutating the key enzymes of the FAS II elongation pathway leads to alterations in DSF turnover as well as turnover of its precursor molecule β‐hydroxyacyl ACP (Figure S5) (Heath & Rock, 1995; White et al., 2005; Zhou et al., 2015). Likewise, it may be possible that RpfF could in turn be playing a role in the turnover of fatty acids for membrane phospholipid biosynthesis, the mechanism of which needs further research and study (Figures S4 and S5).

This study thus reveals the prominent role played by *rpfF* in Xoo in the maintenance of membrane homeostasis through the regulation of LPS, fatty acid, and phospholipid biosynthesis. It also throws light on the difference in regulation by *rpfF* of closely related species of *Xanthomonas*, emphasizing the divergence of function adapted to suit the different host systems that the pathogens infect.

## 4 EXPERIMENTAL PROCEDURES

### 4.1 Bacterial strains, oligonucleotides, plasmids, and growth conditions

The bacterial strains, plasmids, and oligonucleotides used in this study are listed in Tables S2 and S3. Xoo BXO43 and its derived strains were grown at 28°C, maintained on peptone sucrose agar (PSA) plates, or grown in PS broth with shaking at 200 rpm. *E*. *coli* strains were grown at 37°C in Luria‐Bertani (LB) medium with shaking at 200 rpm or on LB agar plates. The antibiotics that were added to the growth medium had the following concentrations: rifampicin (Rif, 50 μg/ml), spectinomycin (Spec, 50 μg/ml), kanamycin (Kan, 50 μg/ml), gentamycin (Gent, 5 μg/ml), nalidixic acid (Nal, 50 μg/ml), and 5‐bromo‐4‐chloro‐3‐indolyl‐d‐galactoside (X‐gal, 25 μg/ml).

### 4.2 Molecular biology

All the molecular biology techniques were performed as described previously (Sambrook et al., 1989). Plasmid DNA was isolated using the Plasmid Midi Kit (Qiagen) according to the manufacturer's instructions. The concentration and purity of the DNA were checked using a Thermo Scientific NanoDrop 2000 spectrophotometer (Thermo Fisher Scientific). PCR was performed using High‐fidelity Phusion *Taq* DNA polymerase (Thermo Fisher Scientific), and restriction digestions and ligations were performed with the respective enzymes (New England Biolabs) according to the manufacturer's instructions. A QIAquick Gel extraction kit (Qiagen) was used for the extraction of DNA from agarose gels. DNA transformations were performed by either heat shock or electroporation.

### 4.3 Construction of *xpsE* and *tolC* deletion mutants in the wild‐type background and the *rpfF* mutant background

XpsE is the ATPase component of the T2SS machinery required for the energy‐dependent secretion of T2SS effectors such as cellulase, lipase, cellobiosidase, and xylanase (Korotkov et al., 2012; Szczesny et al., 2010). TolC is a component of the tripartite AcrAB‐TolC efflux pump that is embedded in the cell envelope and acts as a conduit for the pump. It is responsible for the extrusion of harmful and foreign substances from the periplasm and cytoplasm (Koronakis et al., 2004; Pieretti et al., 2012). Marker‐free deletions of the target genes in the wild‐type Xoo background and the Δ*rpfF* background were made using the suicide vector pK18mobSacB. This vector harbours the kanamycin‐resistance gene (*kan*) cassette and the *sacB* gene, which act as the selection marker and the counterselection marker, respectively (Schäfer et al., 1994). The 5′ and 3′ ends of both the *xpsE* and *tolC* genes were PCR amplified from the Xoo genomic DNA, digested with a common internal restriction enzyme, ligated, and then cloned into the pK18mobSacB vector using the appropriate enzymes to make the deletion construct. The resulting vector constructs (listed in Table S1) were electroporated into the wild‐type Xoo background and the Δ*rpfF* background, and plated on nutrient agar plates supplemented with kanamycin to select for the single recombinants. The single recombinant colonies obtained, which were sucrose‐sensitive and kanamycin‐resistant, were then passaged three times and plated on PSA plates with additional 5% sucrose to select for the double recombinants, which were sucrose‐resistant and kanamycin‐sensitive. The double recombinant colonies obtained were confirmed for the deletion of the respective genes by PCR and sequencing (data not shown) and stored for further study. The single mutant of the *xpsE* gene in the wild‐type background was designated as Δ*xpsE* and the double mutant was designated Δ*xpsE*Δ*rpfF*. Similarly, the *tolC* gene single and double mutants were designated Δ*tolC* and Δ*tolC*Δ*rpfF*.

### 4.4 Complementation of the double mutants Δ*xpsE*Δ*rpfF* and Δ*tolC*Δ*rpfF* with the full‐length *rpfF* gene

The recombinant plasmid, pSC9, with the full‐length *rpfF* gene cloned into the cosmid vector pHM1, was transformed by electroporation into the double mutants Δ*xpsE*Δ*rpfF* and Δ*tolC*Δ*rpfF*. The complemented strains were then selected on PSA plates with spectinomycin, checked for complementation of the phenotypes, and designated as Δ*xpsE*Δ*rpfF*/pSC9 and Δ*tolC*Δ*rpfF*/pSC9, respectively. The wild‐type strain Xoo and its derivatives Δ*rpfF*, Δ*xpsE*, Δ*xpsE*Δ*rpfF*, Δ*tolC*, and Δ*tolC*Δ*rpfF* were then transformed with the empty vector pHM1 by electroporation. These were then designated as Xoo/pHM1, Δ*rpfF*/pHM1, Δ*xpsE*/pHM1, Δ*xpsE*Δ*rpfF*/pHM1, Δ*tolC*/pHM1, and Δ*tolC*Δ*rpfF*/pHM1, and used along with their complements in all the experiments. All experiments performed with these strains had spectinomycin added to the medium for plasmid maintenance.

### 4.5 Determination of the activity of cellulase and lipase

The determination of the cellulase and lipase activity of the different Xoo strains Xoo/pHM1, Δ*rpfF*/pHM1, Δ*rpfF*/pSC9, Δ*xpsE*/pHM1, Δ*xpsE*Δ*rpfF*/pHM1, and Δ*xpsE*Δ*rpfF*/pSC9 were performed as follows. The plate assay for the cellulase activity was performed on PSA plates supplemented with 0.2% carboxymethyl cellulose (CMC) and spectinomycin (Sigma‐Aldrich). Freshly streaked Xoo strains were spotted on the CMC plates in triplicate and incubated at 28°C for 48 h. After this, 2% Congo red in ethanol was added to the plates for staining and they were incubated for 30 min. This was then followed by destaining with 1 M NaCl (Baptista et al., 2010). The halo to colony diameter ratio was subsequently observed and documented. The plate assay for lipase activity was carried out with PS medium supplemented with spectinomycin, 0.5% tributyrin in 100 mM Tris (pH 8), and 25 mM calcium chloride. Freshly streaked strains of Xoo were spotted on tributyrin‐supplemented plates in triplicate, and the halo by colony diameter ratio was observed and documented after incubation at 28°C for 48 h (Rai et al., 2012). The liquid assay for lipase and cellulase was performed using extracellular protein fractions of the culture supernatants of the different Xoo strains, obtained by ammonium sulphate precipitation. The cfu of the Xoo strains was calculated by plating appropriate dilutions of an aliquot of the culture before the separation of the cell pellet and culture supernatant. The cellulase activity was measured by taking 500 μl of appropriately diluted extracellular protein fraction added in a clean glass test tube and heating it for 5 min at 50°C. Five hundred microlitres of 2% CMC in 0.05 M sodium citrate buffer was then added, followed by heating in an incubator at 50°C for 30 min. Three millilitres of dinitrosalicylic acid (DNS) was then added and the mixture vigorously boiled for 5 min in a water bath. The test tubes were then cooled and 20 ml Milli‐Q water was then added followed by mixing achieved by inverting the test tubes several times. The absorbance of each sample was then measured in triplicate at 540 nm (Wood & Bhat, 1988; Zhou et al., 2001) in a multimode spectrophotometer (SpectraMax M5; Molecular Devices). The cellulase activity was determined after cfu normalization using a glucose standard curve, and was represented as ng/ml of glucose released per min per 10^7^ cells. Lipase activity in the extracellular protein fractions was determined as performed earlier, with slight modifications. In this modified method *p*‐nitrophenyl butyrate was used as the substrate instead of *p*‐nitrophenyl laureate; nonetheless, the substrate stock and all the reagents were prepared, and the assay was executed as per the earlier mentioned protocol (Pinsirodom & Parkin, 2001). Fifty microlitres of appropriately diluted extracellular protein fractions was added to 96‐well clear plates in triplicate followed by 100 μl of substrate stock and 100 μl of 0.1 M Tris‐Cl pH 8. This was then incubated for 1 h and the absorbance was measured at 410 nm. The lipase activity was then determined using a *p*‐nitrophenol standard curve and calculated after cfu normalization as μmol of *p*‐nitrophenol released per minute per 10^7^ cells (Pinsirodom & Parkin, 2001).

### 4.6 Isolation of extracellular protein fractions from culture supernatants

The Xoo strains Xoo/pHM1, Δ*rpfF*/pHM1, Δ*rpfF*/pSC9, Δ*xpsE*/pHM1, Δ*xpsE*Δ*rpfF*/pHM1, and Δ*xpsE*Δ*rpfF*/pSC9 were grown in 100 ml of PS medium until they reached an early log phase with an OD_600_ between 0.4 and 0.6. A 1 ml aliquot of these cultures was kept aside for dilution plating to obtain the cfu of each strain, and the remaining culture was centrifuged at 17,000 × *g* for 30 min to separate the cell pellet from the culture supernatant. Extracellular protein fractions were then isolated from the supernatants by 50% (wt/vol) ammonium sulphate precipitation, followed by centrifugation at 17,000 × *g* for 30 min (Ray et al., 2000). The extracellular protein precipitate thus obtained was then dissolved in 2 ml of 50 mM ammonium acetate buffer pH 5.2 and dialysed in 10 mM Tris‐Cl pH 8. The protein content of the extracellular fractions was then determined by Bradford's method, aliquoted in smaller volumes, and stored at −80°C for further studies. The same procedure was followed for the isolation of extracellular protein fractions from Xoo strains that were grown in PS supplemented with 5% sucrose.

### 4.7 SDS‐PAGE and western blot analysis

The cell number‐normalized protein fractions were separated by 12% SDS‐PAGE along with either unstained (for silver staining) or prestained (for western blot) protein markers (Thermo Fisher), as described earlier (Rai et al., 2012). The visualization of the gels was done by silver nitrate staining (Sambrook et al., 1989). The western blot analysis was done with anticellulase and antilipase antibodies (Rai et al., 2012). The secondary antibody was goat antirabbit immunoglobulin G conjugated to horseradish peroxidase (HRP) (1:2000 dilution) obtained from Sigma‐Aldrich. Detection was performed using an ECL kit (Amersham ECL plus western blotting detection reagents; GE Healthcare).

### 4.8 Determination of EGFP in the extracellular and whole‐cell protein fractions of Xoo strains

The Xoo strains were transformed with the plasmid pMP2464, bearing the EGFP coding sequence, by electroporation and the transformants were selected on PS plates supplemented with spectinomycin and gentamycin (Pradhan et al., 2012). These transformants were then grown until early log phase, followed by serial dilution plating for cfu determination and extraction of extracellular protein fractions as described earlier. To isolate the whole‐cell protein fractions, the pellet obtained was resuspended in 50 mM acetate buffer (pH 5.4) with 0.2 μg/ml lysozyme, and the cell suspension was left in ice for 30 min. This was followed by the addition of 1 mM phenylmethylsulfonyl fluoride (PMSF) to the suspension, and sonication for 30 min at a medium pulse rate of the 30 s on and 45 s off using a BioruptorTM UCD‐200 (Diagenode) under ice‐cold conditions. The extracellular protein fractions were then resolved by SDS‐PAGE and probed for EGFP by western blotting as described earlier.

### 4.9 Determination of alkaline phosphatase activity in the extracellular protein fractions

Alkaline phosphatase in both the extracytoplasmic as well as the whole‐cell protein fractions was determined as described earlier (Sikora et al., 2007) using *p*‐nitrophenol phosphate (Sigma Aldrich) as the substrate, and the activity was calculated as μmol of *p*‐nitrophenol released per minute per 10^7^ cells. The activity in the supernatants was depicted as a percentage of the enzyme activity in the extracytoplasmic fraction compared to the total activity in the extracytoplasmic and the whole‐cell protein fractions.

### 4.10 Assay for the accumulation of NPN and EtBr

Overnight cultures of the Xoo strains Δ*tolC*/pHM1, Δ*tolC*Δ*rpfF*/pHM1, and Δ*tolC*Δ*rpfF*/pSC9 were used to inoculate fresh PS medium with the respective antibiotics and the cultures were grown to early log phase. These cultures were then centrifuged at 4303 × *g* for 10 min and washed twice with assay buffer (5 mM HEPES [4‐(2‐hydroxyethyl)‐1‐piperazineethanesulfonic acid] pH 7.2 with 137 mM NaCl). The washed cells were then resuspended in the assay buffer and the cell numbers were normalized to approximately 10^9^ cells. An aliquot of these cells was taken, serially diluted, and plated on PS agar plates for the determination of cfu. One hundred microlitres of the cells was added in triplicate to a 96‐well plate followed by the addition of 100 μl of 20 μM NPN (Sigma Aldrich) in assay buffer. Buffer with no added NPN as well as only NPN with no added cells were kept as controls. Immediately after the addition of NPN, the fluorescence was measured every 25 s for 10 min at an excitation wavelength of 355 nm and an emission wavelength of 402 nm (Helander & Mattila‐Sandholm, 2000). Fluorescence values were normalized with the cfu and the Δ*tolC*/pHM1 fluorescence at time 0 was defined as 100% (Zou et al., 2012). The EtBr uptake assay was performed as described earlier (Zou et al., 2012) with a few modifications. Phosphate‐buffered saline (PBS) pH 7.3 was used as the buffer and the Xoo strains Δ*tolC*/pHM1, Δ*tolC*Δ*rpfF*/pHM1, and Δ*tolC*Δ*rpfF*/pSC9, grown to the early‐log phase, were assayed. These strains were washed twice and suspended in PBS to obtain an OD_600_ of 0.4. An aliquot of these cells was taken, serially diluted, and plated on PS agar plates for the determination of cfu. One hundred microlitres of the cells was added in triplicate to a 96‐well plate followed by the addition of 100 μl of 12 μM EtBr (Research Products International) in assay buffer. Buffer with no added EtBr as well as only EtBr with no added cells were kept as controls (Paixão et al., 2009). Immediately after the addition of EtBr, the fluorescence was measured every 25 s for 10 min at an excitation wavelength of 545 nm and an emission wavelength of 600 nm. Fluorescence values were normalized with the cfu and the wild‐type Xoo fluorescence at time 0 was defined as 100%.

### 4.11 Determination of sensitivity of the Xoo strains to detergents

The Xoo strains Xoo/pHM1, Δ*rpfF*/pHM1, and Δ*rpfF*/pSC9 were grown until the late log phase. They were then normalized to an OD_600_ of 0.8 after which the cells were serially diluted in PBS pH 7.3. An aliquot (2.5 μl) of these serially diluted cells were then spotted in triplicate onto PSA with spectinomycin and additionally onto PS medium supplemented with spectinomycin and Tween 20, Triton X 100, or SDS (Sigma‐Aldrich) (Muheim et al., 2017; Parkin et al., 2015; Zou et al., 2012). The plates were then kept at 28°C for 48 h and the difference in growth due to the presence of detergents was then compared with that of growth in PSA alone.

### 4.12 Phospholipid analysis of Xoo and *E*. *coli* strains

The Xoo strains Xoo/pHM1, Δ*rpfF*/pHM1, and Δ*rpfF*/pSC9, the *E*. *coli* WT strain, and *E. coli* LPS mutant strains JW3596 and JW3597 (Baba et al., 2006; Parsons & Rock, 2013) were grown until the late log phase and normalized to an OD_600_ of 0.8. The extraction of the phospholipids was done by first treating the OD‐normalized bacterial cultures to 12.5 mM of sodium acetate of pH 4.4 at 100°C for 30 min. This was then followed by the extraction of the lipids in Bligh and Dyer mixture (Li et al., 2010). At the end of each extraction, the lipid samples were lyophilized and then dissolved in 100 μl of 2:1 chloroform:methanol mixture. Ten microlitres of these samples along with the *E*. *coli* total lipid standard (Sigma‐Aldrich) was then spotted on the TLC plates. The TLC plates were developed in chloroform, methanol, water, and ammonia solvent (65:25:3:6:0.4, vol/vol/vol/vol). Phospholipids on the TLC plate were then visualized by spraying with 10% sulphuric acid in ethanol followed by charring at 200°C.

### 4.13 LPS extraction of Xoo and *E*. *coli* strains

The strains Xoo/pHM1, Δ*rpfF*/pHM1, Δ*rpfF*/pSC9, and the *E*. *coli* WT strain, and *E. coli* LPS mutant strains JW3596 and JW3597 were grown until the late log phase and then normalized to an OD_600_ of 1.3. LPS from 1 ml pellets of these OD‐normalized strains were then extracted by the hot aqueous phenol method as described previously (Davis & Goldberg, 2012). DNase I and RNase were used to rid the samples of nucleic acids and the samples were incubated for 3 h with proteinase K for the removal of protein contaminants. The samples were then heated at 65°C for 15 min in Tris‐saturated phenol and then LPS extracted from the lower layer after centrifugation. Fifteen microlitres of the extracted samples was then loaded onto 12% SDS‐acylamide gel for SDS‐PAGE then visualized by silver staining.

### 4.14 GC/MS analysis of cellular fatty acids

The total cell fatty acid of the wild‐type Xoo/pHM1, Δ*rpfF*/pHM1, and the complemented strain Δ*rpfF*/pSC9 were extracted by a modification of the Bligh and Dyer protocol (Sasser, 1990), wherein the cells were grown on PSA plates and around 40 mg of the cells were harvested from the third quadrant of the plate and placed in clean glass tubes. These cells were then subjected to saponification with sodium hydroxide solution and methylation with 6 M HCl and methanol. The extraction was done in methyl *tert*‐butyl ether followed by sample clean‐up with 0.3 M sodium hydroxide, after which the samples were subjected to GC/MS analysis on a GC‐2010 Plus system (Shimadzu Corporation). A SH‐RtxTM‐65 mid‐polarity phase column with 65% diphenyl/35% methyl polysiloxane was used and the temperature of the column was ramped from 170 to 270°C at 5°C/min. Helium was used as the carrier gas at a constant flow rate of 0.8 ml/min. The mass spectrometer was operated in the electron impact mode at 70 eV with a scan range of 50–500 *m*/*z*. The acquired spectrum was searched against the standard National Institute of Standards and Technology (NIST) mass spectral library.

### 4.15 Expression analysis by RT‐qPCR

The wild type and the *rpfF* mutant were grown in PS supplemented with spectinomycin. RNA was then isolated from the cells harvested in the mid‐log, late‐log, and stationary phases. The TRIzol (Invitrogen) method was used for the isolation of total RNA according to the manufacturer's instructions. cDNA was synthesized with SuperScript III reverse transcriptase (Invitrogen), and qPCR was performed using 2× DyNAmo Color Flash SYBR Green (Thermo Scientific) following the manufacturer's instructions in a 7500 real‐time PCR system (Applied Biosystems) as described previously (Chatterjee et al., 2008). Analysis was done by relative quantification software (Applied Biosystems). The primers used for RT‐qPCR are listed in Table S2.

### 4.16 Virulence assay and in planta survivability and migration assay

The wound inoculation method (Kauffman et al., 1973) was used for the infection of 40‐to 45‐day‐old susceptible rice plants (cv. Taichung Native 1). Xoo strains were grown until saturation; suspensions normalized to approximately 10^9^ cells/ml were used for clip inoculation. Around 25 leaves per experiment were clipped per strain and the average lesion length was measured 14 days postinoculation. This experiment was repeated three times and the mean values were then plotted, with error bars representing the standard error of the mean. In planta survival and migration assays were performed as described previously (Ji et al., 2018; Verma et al., 2018).

### 4.17 Statistical analysis

Graphs, calculations, and statistical analyses were performed using GraphPad Prism software v. 8.0 for Windows (GraphPad Software). A paired two‐tailed Student's *t* test was used for pairwise comparisons. An ordinary one‐way analysis of variance with post hoc analysis (Tukey's honestly significant difference [HSD] test) was applied to data from three or more groups. *p* values of <0.05 and a fold change of >2 were considered statistically significant.

## Supporting information


**FIGURE S1** *rpfF* mutant of *Xanthomonas oryzae* pv. *oryzae* (Xoo) is significantly permeable to 1‐*N*‐phenylnapthylamine (NPN) and ethidium bromide (EtBr). TheTolC mutant *∆tolC*/pHM1, the TolC‐*rpfF* double mutant *∆tolC ∆rpfF*/pHM1, and the double mutant with the full‐length *rpfF* gene *∆tolC ∆rpfF*/pSC9 were checked for the uptake of (a) EtBr and (b) NPN. The experiments were performed as three biological triplicates. Vertical error bars represent *SD*. ***p < 0.001 of TolC‐*rpfF* double mutant to TolC single mutant as determined by a one‐way analysis of variance followed by post hoc Tukey HSD analysis at the 600th second

**FIGURE S2** *Xanthomonas oryzae* pv. *oryzae* (Xoo) *rpfF* mutant shows higher sensitivity to detergents. The strains Xoo/pHM1, Δ*rpfF*/pHM1, and Δ*rpfF*/pSC9 were grown to log phase and normalized to an OD_600_ of 0.8 after which they were serially diluted and spotted on PSA plates supplemented with (a) spectinomycin, (b) spectinomycin and 0.1% Tween 20, (c) spectinomycin and 0.2% Triton X‐100, and (d) spectinomycin and 0.01% SDS. The experiment was performed in three biological replicates

**FIGURE S3** The expression of *rpfB* in the *Xanthomonas oryzae* pv. *oryzae* (Xoo) *rpfF* mutant is significantly up‐regulated. Relative quantification of *rpfB* gene by quantitative reverse transcription‐PCR (RT‐qPCR). RNA was isolated from the strains by the TRIzol method. cDNA was synthesized with the GoScript reverse transcription system (Promega) and RT‐qPCR was performed using GoTaq SYBR green (Promega) following the manufacturer’s instructions in a 7500 real‐time PCR system. The analysis was done by SDS relative quantification software (Applied Biosystems) and the output data were analyzed by fold change calculation (2^−ΔΔ^ *^C^* ^t^) with respect to the wild‐type strain, using 16S rRNA as the endogenous control. Standard errors were calculated based on three independent experiments. Paired Student’s *t* test was done to determine the significant difference of the test strain with wild‐type Xoo. ***p ≤ 0.001, error bars represent standard deviation

**FIGURE S4** Proposed model for the antagonistic mechanism of membrane homeostasis by *rpfF* in *Xanthomonas campsetrsi* pv. *campestris* (Xcc) and *Xanthomonas oryzae* pv. *oryzae* (Xoo). In Xcc, the unregulated RpfF thioesterase activity in the *rpfB rpfC* double mutant leads to uninhibited cleavage of acyl ACP intermediates, resulting in the release of free fatty acids in the extracellular medium, which eventually causes cell membrane damage. This uninhibited thioesterase activity of RpfF has been proposed to be counteracted by the fatty acyl‐CoA ligase (FCL) activity of RpfB in the wild‐type strain. It does this by sequestering free fatty acids back into the cell to produce acyl‐CoAs, which are probably rerouted back to the membrane phospholipid biosynthesis pathway for the maintenance of membrane integrity. In Xoo lack of RpfF activity causes reduced cellular fatty acid and altered phospholipid, and LPS profile, which could be the likely cause for the hyper‐release of the T2SS effectors and intracellular proteins in the *rpfF* mutant. *rpfB* expression and possibly its FCL activity in Xoo are regulated by the quorum‐sensing molecule DSF, which could be one of the plausible explanations for lack of membrane stability in the DSF synthase *rpfF* mutant. Other likely explanations for the presence of a compromised membrane in *rpfF* mutant could be regulation of the FAS II pathway and/or the LPS biosynthesis pathway

**FIGURE S5** Alteration in the expression of key players of the FAS II elongation module causes significant changes in DSF levels. (a) Blocking of FabB and FabF, the β‐keto‐acyl‐ACP‐synthetase of FAS II in *Xanthomonas campestris* pv. *campestris*, causes reduction in DSF concentration whereas (b) overexpression of FabG‐ the β‐keto‐acyl‐ACP‐reductase causes increase in DSF production. (c) β‐hydroxy‐acyl‐ACP‐ one of the intemediates of the FAS II pathway is the precursor molecule for DSF synthesis. The arrowhead of the outer arrows indicates the equilibrium positions of the enzymatic reaction

**TABLE S1** Comparison of the fatty acid profiles in the different bacterial strains

**TABLE S2** Strains and plasmids used in this study

**TABLE S3** Oligonucletides used in this study

## Data Availability

The data that supports the findings of this study are available in the supplementary material of this article and also are available from the corresponding author upon reasonable request.
