# Supplementary figures and images for: The diffusible signal factor synthase, RpfF, in Xanthomonas oryzae pv. oryzae is required for the maintenance of membrane integrity and virulence
Source: Mol Plant Pathol. 2021 Oct 26;23(1):118–32. doi: 10.1111/mpp.13148 (PMC8659556; doi:10.1111/mpp.13148)

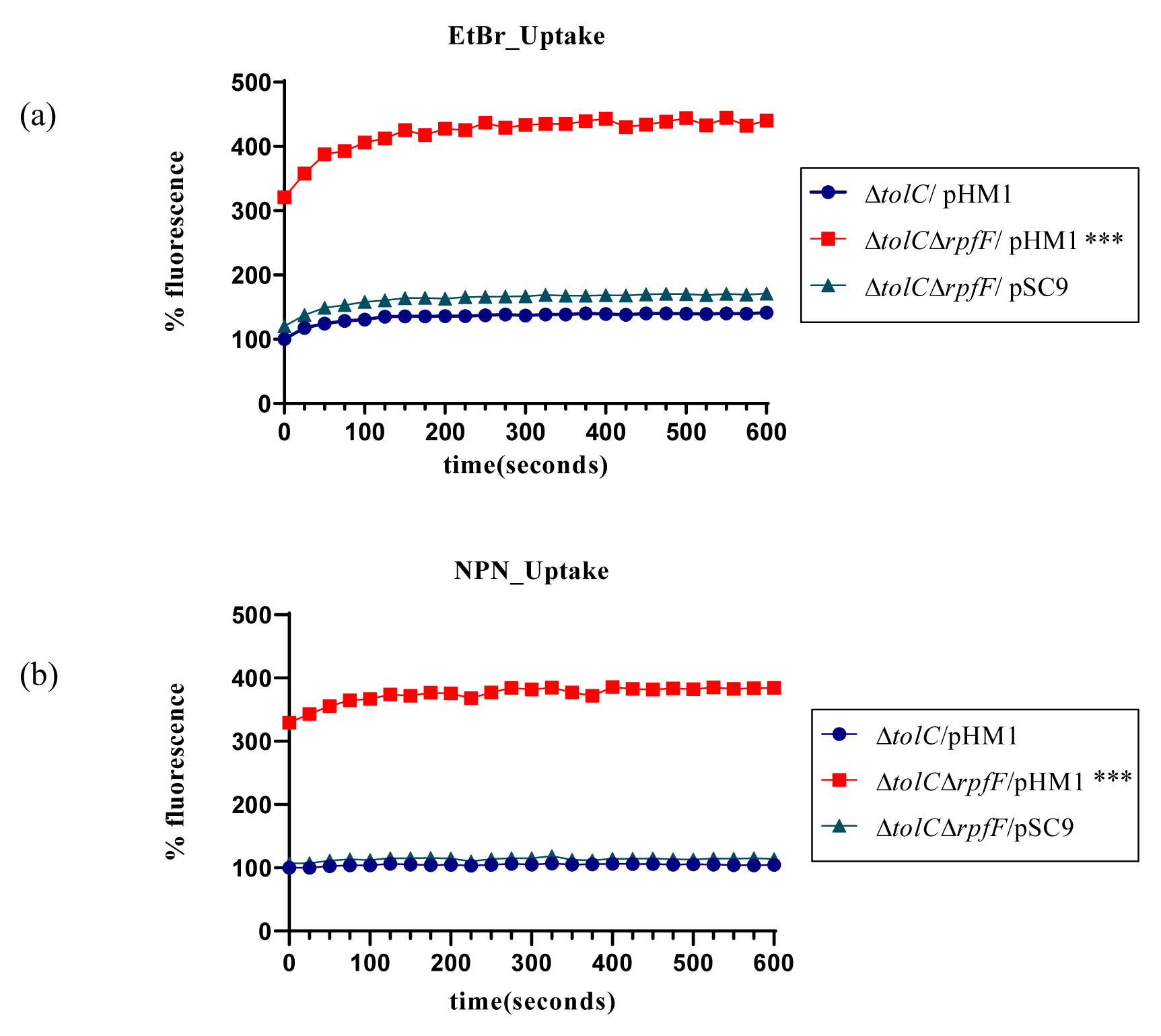

Supplement: Supplementary file 1 — FIGURE S1 rpfF mutant of Xanthomonas oryzae pv. oryzae (Xoo) is significantly permeable to 1‐N‐phenylnapthylamine (NPN) and ethidium bromide (EtBr). TheTolC mutant ∆tolC/pHM1, the TolC‐rpfF double mutant ∆tolC ∆rpfF/pHM1, and the double mutant with the full‐length rpfF gene ∆tolC ∆rpfF/pSC9 were checked for the uptake of (a) EtBr and (b) NPN. The experiments were performed as three biological triplicates. Vertical error bars represent SD. ***p < 0.001 of TolC‐rpfF double mutant to TolC single mutant as determined by a one‐way analysis of variance followed by post hoc Tukey HSD analysis at the 600th second [file MPP-23-118-s004.tif]

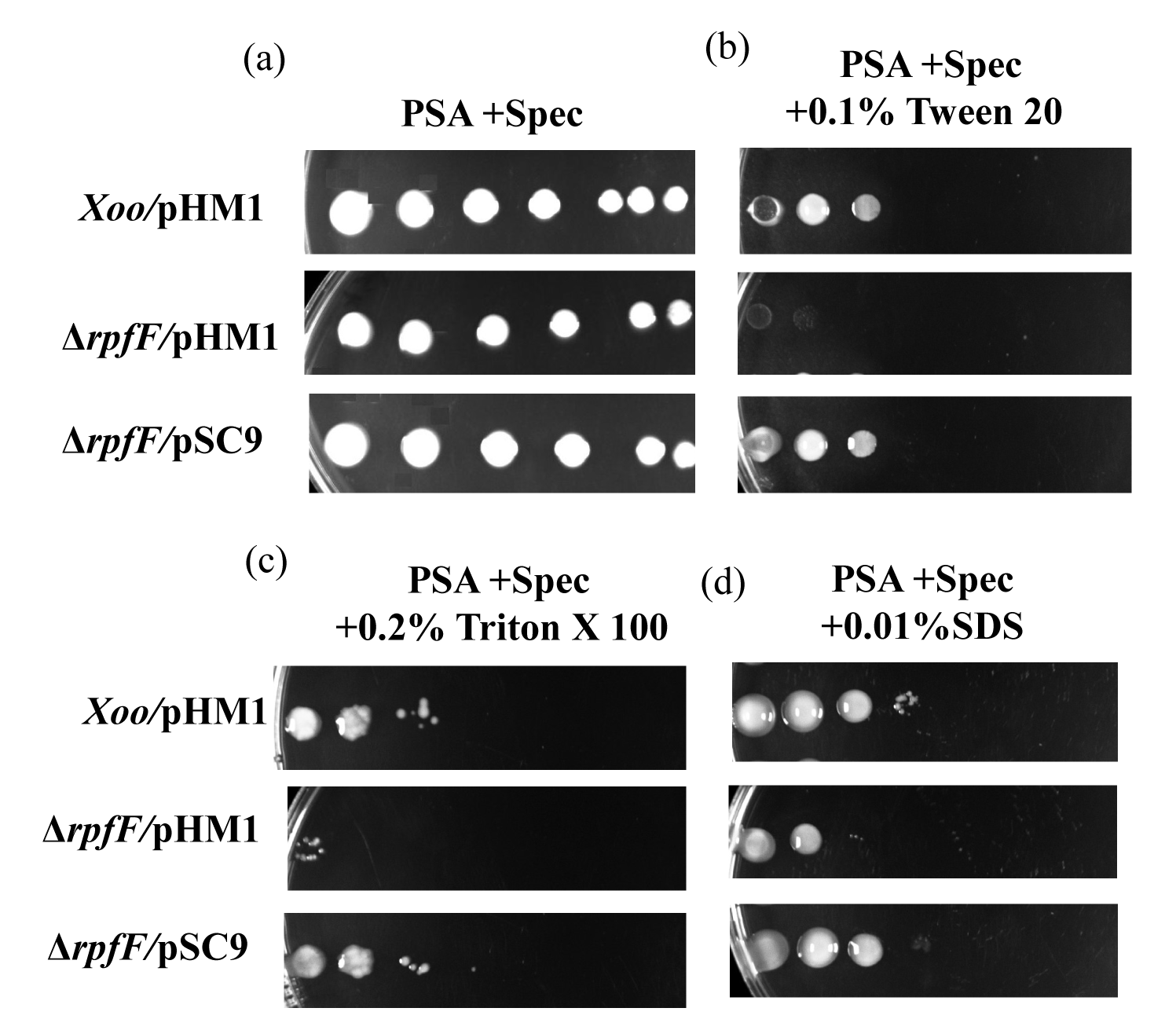

Supplement: Supplementary file 2 — FIGURE S2 Xanthomonas oryzae pv. oryzae (Xoo) rpfF mutant shows higher sensitivity to detergents. The strains Xoo/pHM1, ΔrpfF/pHM1, and ΔrpfF/pSC9 were grown to log phase and normalized to an OD600 of 0.8 after which they were serially diluted and spotted on PSA plates supplemented with (a) spectinomycin, (b) spectinomycin and 0.1% Tween 20, (c) spectinomycin and 0.2% Triton X‐100, and (d) spectinomycin and 0.01% SDS. The experiment was performed in three biological replicates [file MPP-23-118-s003.tif]

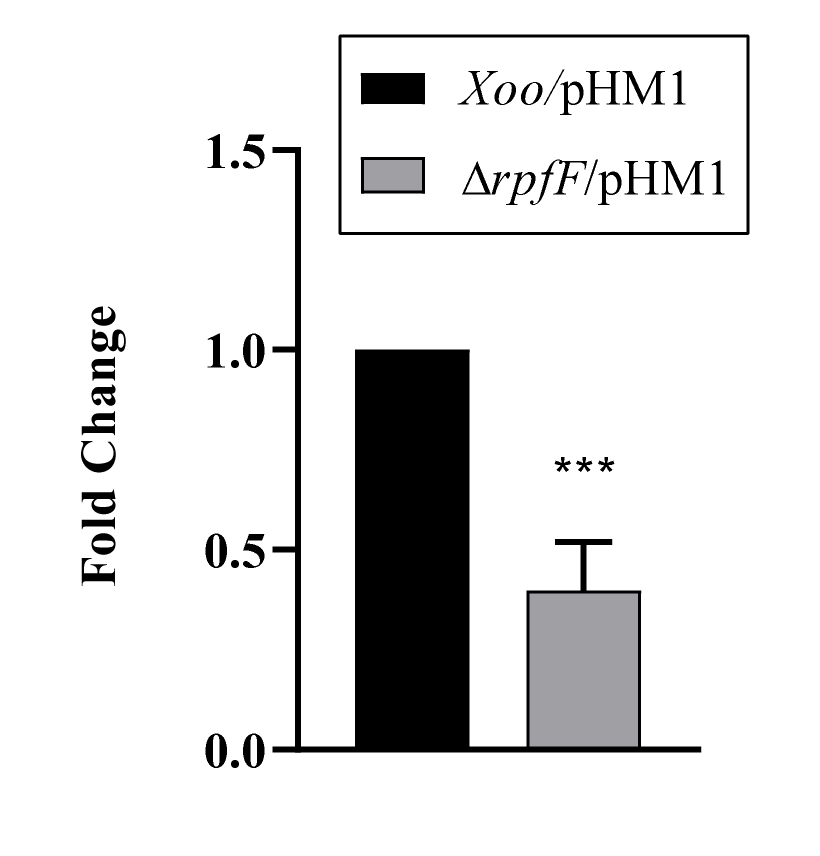

Supplement: Supplementary file 3 — FIGURE S3 The expression of rpfB in the Xanthomonas oryzae pv. oryzae (Xoo) rpfF mutant is significantly up‐regulated. Relative quantification of rpfB gene by quantitative reverse transcription‐PCR (RT‐qPCR). RNA was isolated from the strains by the TRIzol method. cDNA was synthesized with the GoScript reverse transcription system (Promega) and RT‐qPCR was performed using GoTaq SYBR green (Promega) following the manufacturer’s instructions in a 7500 real‐time PCR system. The analysis was done by SDS relative quantification software (Applied Biosystems) and the output data were analyzed by fold change calculation (2−ΔΔ C t) with respect to the wild‐type strain, using 16S rRNA as the endogenous control. Standard errors were calculated based on three independent experiments. Paired Student’s t test was done to determine the significant difference of the test strain with wild‐type Xoo. ***p ≤ 0.001, error bars represent standard deviation [file MPP-23-118-s005.tif]

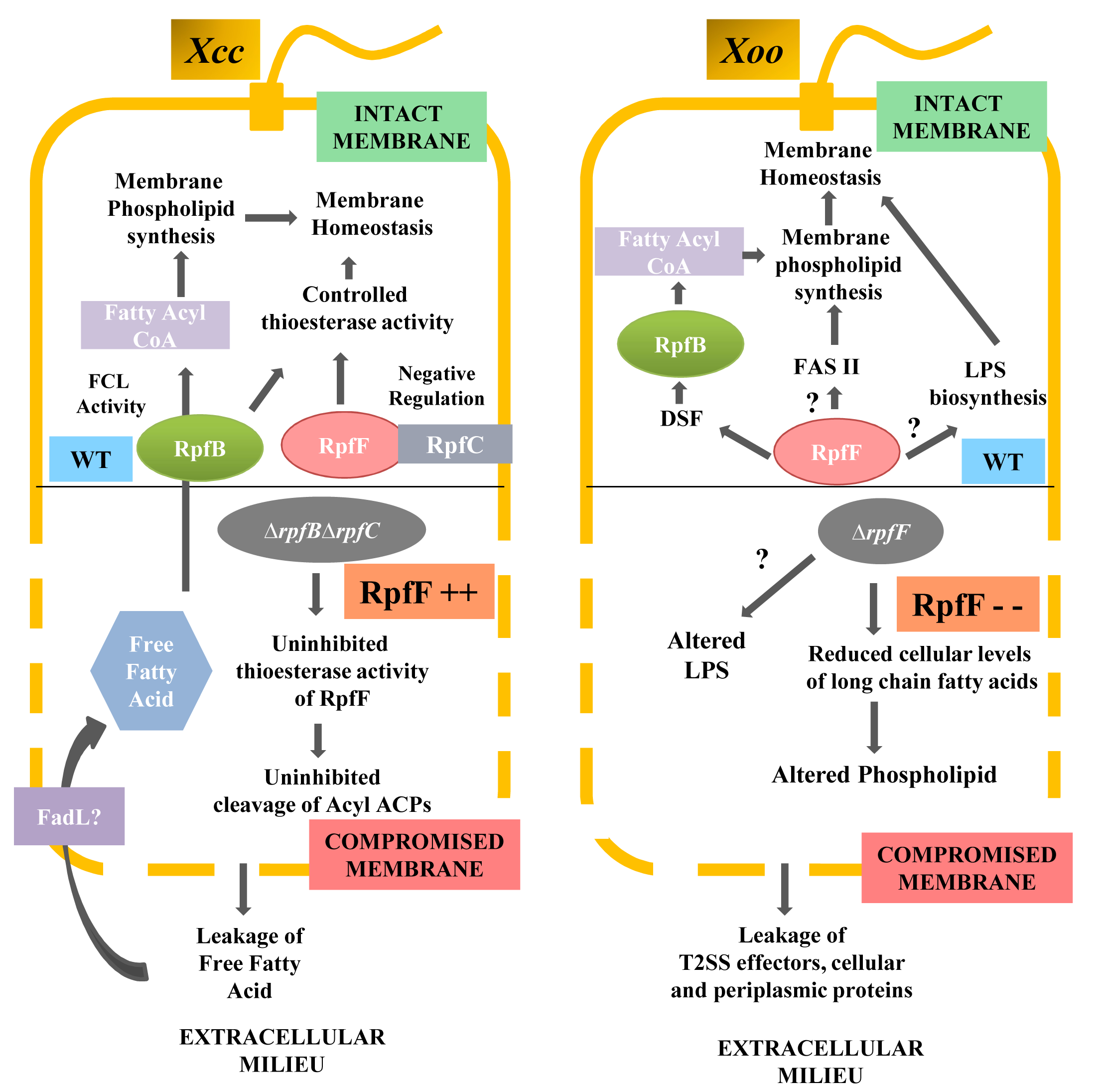

Supplement: Supplementary file 4 — FIGURE S4 Proposed model for the antagonistic mechanism of membrane homeostasis by rpfF in Xanthomonas campsetrsi pv. campestris (Xcc) and Xanthomonas oryzae pv. oryzae (Xoo). In Xcc, the unregulated RpfF thioesterase activity in the rpfB rpfC double mutant leads to uninhibited cleavage of acyl ACP intermediates, resulting in the release of free fatty acids in the extracellular medium, which eventually causes cell membrane damage. This uninhibited thioesterase activity of RpfF has been proposed to be counteracted by the fatty acyl‐CoA ligase (FCL) activity of RpfB in the wild‐type strain. It does this by sequestering free fatty acids back into the cell to produce acyl‐CoAs, which are probably rerouted back to the membrane phospholipid biosynthesis pathway for the maintenance of membrane integrity. In Xoo lack of RpfF activity causes reduced cellular fatty acid and altered phospholipid, and LPS profile, which could be the likely cause for the hyper‐release of the T2SS effectors and intracellular proteins in the rpfF mutant. rpfB expression and possibly its FCL activity in Xoo are regulated by the quorum‐sensing molecule DSF, which could be one of the plausible explanations for lack of membrane stability in the DSF synthase rpfF mutant. Other likely explanations for the presence of a compromised membrane in rpfF mutant could be regulation of the FAS II pathway and/or the LPS biosynthesis pathway [file MPP-23-118-s001.tif]

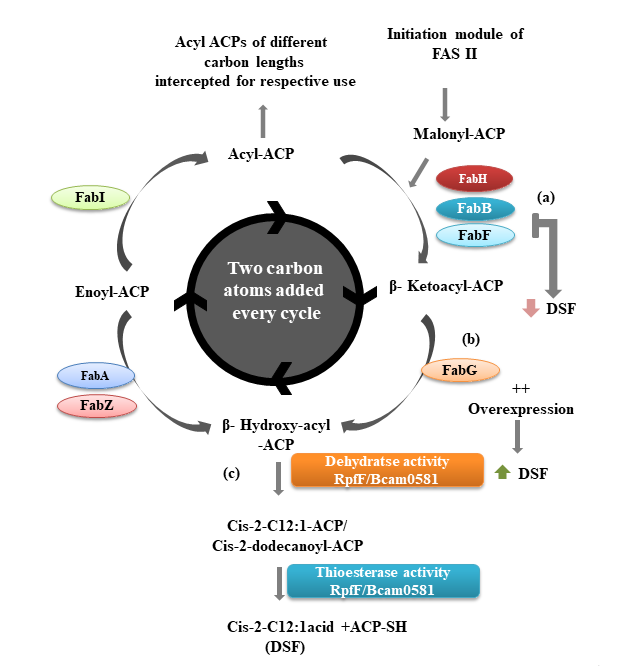

Supplement: Supplementary file 5 — FIGURE S5 Alteration in the expression of key players of the FAS II elongation module causes significant changes in DSF levels. (a) Blocking of FabB and FabF, the β‐keto‐acyl‐ACP‐synthetase of FAS II in Xanthomonas campestris pv. campestris, causes reduction in DSF concentration whereas (b) overexpression of FabG‐ the β‐keto‐acyl‐ACP‐reductase causes increase in DSF production. (c) β‐hydroxy‐acyl‐ACP‐ one of the intemediates of the FAS II pathway is the precursor molecule for DSF synthesis. The arrowhead of the outer arrows indicates the equilibrium positions of the enzymatic reaction [file MPP-23-118-s002.tif]
