# Supplementary material for: The diffusible signal factor synthase, RpfF, in Xanthomonas oryzae pv. oryzae is required for the maintenance of membrane integrity and virulence
Source: Mol Plant Pathol. 2021 Oct 26;23(1):118–32. doi: 10.1111/mpp.13148 (PMC8659556; doi:10.1111/mpp.13148)
Supplement: Supplementary file 6 — TABLE S1 Comparison of the fatty acid profiles in the different bacterial strains [file MPP-23-118-s008.doc]

**Table S1.** Comparison of the fatty acid profiles in the different bacterial strains. Data derived from Total Ion Chromatogram shown in Figure 7. ND – not detected.

|  |  |  | PERCENTAGE | | |
| --- | --- | --- | --- | --- | --- |
| RETENTION TIME (min) | CELLULAR FATTY ACID | SHORT HAND | *Xoo/*pHM1 | ∆*rpfF/*pHM1 | ∆*rpfF/*pSC9 |
| 3.626 | Methyl-6-methyl heptanoic acid | 6-Me-7:0 | 4.29 | 24.34 | 2.78 |
| 4.194 | 4methyl-2-propyl-pentanoic acid | 4-Me-2-Propyl-5:0 | 3.47 | 13.3 | 1.43 |
| 4.385 | 3,5-dimethyl-heptanoic acid | 3,5-diMe-7:0 | 5.99 | 38.67 | 3.49 |
| 11.87 | 8-hydroxy-octanoic acid | 8-OH-8:0 | 6.89 | 9.53 | 3.86 |
| 14.61 | 10-hydroxy- decanoic acid | 10-Me-11:0 | 29.71 | ND | 31.84 |
| 14.61 | 9-hydroxy-nonanoic acid | 9-OH-9:0 | 31.1 | ND | 33.1 |
| 15.38 | 9-hydroxy-octanoic acid | 9-OH-10:0 | 0.35 | ND | 0.21 |
| 15.85 | 9-bromo-nonanoic acid | 9-Br-9:0 | 7.03 | ND | 7 |
