# Supplementary material for: The diffusible signal factor synthase, RpfF, in Xanthomonas oryzae pv. oryzae is required for the maintenance of membrane integrity and virulence
Source: Mol Plant Pathol. 2021 Oct 26;23(1):118–32. doi: 10.1111/mpp.13148 (PMC8659556; doi:10.1111/mpp.13148)
Supplement: Supplementary file 7 — TABLE S2 Strains and plasmids used in this study [file MPP-23-118-s006.doc]

**Table S2. Strains, plasmids used in this study**

| **Strains** | **Descriptions** | **Reference** |
| --- | --- | --- |
| DH5α | F′/ endA1 hsdR17 (rk– mk+) supE44 thi-1 recA1 gyrA relA1 f80dlacZDM15 (lacZYA-argF) U169 | Lab collection |
| JW3596-1 | F-, *Δ(araD-araB)567*, *ΔlacZ4787*(::rrnB-3), *λ^-^*, *ΔrfaC733::kan*, *rph-1*, *Δ(rhaD-rhaB)568*, *hsdR514* | Baba et al., 2006 |
| JW3597-1 | F-, *Δ(araD-araB)567*, *ΔlacZ4787*(::rrnB-3), *λ^-^*, *ΔrfaL734::kan*, *rph-1*, *Δ(rhaD-rhaB)568*, *hsdR514* | Baba et al., 2006 |
| *X*. *oryzae* pv. *oryzae* (BXO43) | WT *rif*-2; Rif^r^ | Lab collection |
| Δ*rpfF* | In frame deletion of *rpfF*; derivative of BXO43; Rif^r^ | Lab collection |
| Δ*rpf* (pSC9) | *ΔrpfF* mutant harboring the complementing plasmid pSC9; derivative of BXO43; rpfF+ ; Rif^r^ Spec^r^ | Lab collection |
| Δ*xpsE* | In frame deletion of *xpsE*; derivative of BXO43; Rif^r^ | This study |
| Δ*xpsE* Δ*rpfF* | In frame deletion of *xpsE* in Δ*rpfF*; derivative of BXO43; Rif^r^ | This study |
| Δ*xpsE* Δ*rpfF*/pSC9 | Δ*xpsE* Δ*rpfF* double mutant harbouring the complementing plasmid pSC9; derivative of BXO43; rpfF+ ; Rif^r^ Spec^r^ | This study |
| Δ*tolC* | In frame deletion of *tolC*; derivative of BXO43; Rif^r^ | This study |
| Δ*tolC*Δ*rpfF* | In frame deletion of *tolC* in Δ*rpfF*; derivative of BXO43; Rif^r^ | This study |
| Δ*tolC* Δ*rpfF*/pSC9 | Δ*tolC* Δ*rpfF* double mutant harbouring the complementing plasmid pSC9; derivative of BXO43; rpfF+ ; Rif^r^ Spec^r^ | This study |
| *xadM*::pK18mob/pHM1 | *xadM2*::pK18mobApr *rif*-2; Apr ; Vir– derivative of BXO43 | Pradhan et al., 2012 |
| **Plasmids** | **Descriptions** | **Reference** |
| pK18mobsacB | Km^r^ pUC18 derivative; lacZα mobs site sacB | Schäfer et al., 1994 |
| pHM1 | Broad-host-range cosmid vector, pSa *ori*, Spec^r^ | Innes *et al*., 1988 |
| pMP2464 | pBBR1MCS5 derivative; EGFP+, Gm^r^ | DeFeyter et al. 1990 |
| pPS1 | pK18mobsacB with 504bp PCR fragment of *xpsE* 5’ end and  a 639bp PCR fragment of *xpsE* 3’ end from the BXO43  genome; Kan^r^ | This study |
| pPS2 | pK18mobsacB with 319bp PCR fragment of *tolC* 5’ end and  a 481bp PCR fragment of *tolC* 3’ end from the BXO43  genome; Kan^r^ | This study |
| pSC9 | pHM1 harbouring full length *rpfF* gene from BXO43 | Lab collection |

Rif^r^ , Spec^r^ , Kan^r^ and Gm^r^ refer to resistance to the antibiotics Rifampicin ,Spectinomycin , Kanamycin and Gentamycin respectively.

Baba, T., Ara, T., Hasegawa, M., Takai, Y., Okumura, Y., Baba, M., ... & Mori, H. (2006). Construction of Escherichia coli K‐12 in‐frame, single‐gene knockout mutants: the Keio collection. *Molecular systems biology*, 2, 2006-0008.

DeFeyter, R., Kado, C. I., & Gabriel, D. W. (1990). Small, stable shuttle vectors for use in Xanthomonas, *Gene*, 88, 65-72.

Innes RW., Hirose MA., & Kuempel PL. (1988). Induction of nitrogen-fixing nodules on clover requires only 32 kilobase pairs of DNA from Rhizobium trifolii symbiosis plasmid. *Journal of Bacteriol*ogy, 170, 3793–3802.

Pradhan, B.B., Ranjan, M. and Chatterjee, S. (2012) XadM, a novel adhesin of *Xanthomonas oryzae pv. oryzae*, exhibits similarity to Rhs family proteins and is required for optimum attachment, biofilm formation, and virulence. *Molecular Plant Microbe Interactions*, **25**, 1157–1170.

Schäfer, A., Tauch, A., Jäger, W., Kalinowski, J., Thierbach, G., & Pühler, A. (1994). Small mobilizable multi-purpose cloning vectors derived from the *Escherichia coli* plasmids pK18 and pK19: selection of defined deletions in the chromosome of *Corynebacterium glutamicum.* *Gene*, 145, 69-73.
