# Supplementary material for: The diffusible signal factor synthase, RpfF, in Xanthomonas oryzae pv. oryzae is required for the maintenance of membrane integrity and virulence
Source: Mol Plant Pathol. 2021 Oct 26;23(1):118–32. doi: 10.1111/mpp.13148 (PMC8659556; doi:10.1111/mpp.13148)
Supplement: Supplementary file 8 — TABLE S3 Oligonucletides used in this study [file MPP-23-118-s007.doc]

**Table S3.** Oligonucletides used in this study

| **Oligonucletides** | **Sequence** | **Reference** |
| --- | --- | --- |
| ∆*xpsE* F1 XbaI | GATCTAGAGCGTGCGGTTGATCGTCC | This study |
| ∆*xpsE* R1 XhoI | CCCTCGAGGATTTCCGAGCGCAAGCC | This study |
| ∆*xpsE* F2 XhoI | CCCTCGAGAACGCCCTACGCAGCATC | This study |
| ∆*xpsE* R2 Hind III | GGAAGCTTGCGGATTGATGTGCTGGC | This study |
| xpsEoutF | ATCAAGTTGGTCTGTGCGGCGAGCTAC | This study |
| xpsEoutR | TCGGGAATCGGGACTTGGGAATGGGCA | This study |
| ∆*tolC* F1 ECoRI | GGGAATTCCGCACGCGCCAATCTGGACCG | This study |
| ∆*tolC* R1XbaI | GCTCTAGAGTGGATCATTCAGAACTGGAA | This study |
| ∆*tolC* F2XbaI | GCTCTAGAACCGACTTGCAGGATGTGAAT | This study |
| ∆*tolC* R2 HindIII | GGAAGCTTCTGGTGCAGGTCGGCGCGGAT | This study |
| *rpoE* RT F | TGATGAATGACACCGACACC | This study |
| *rpoE* RT R | TCCAGAAGCAATCGTTGATG | This study |
| *mucD* RT F | GAGGCAACCTGATGAACCAT | This study |
| *mucD* RT R | TGATGGTGGTTTCGACATTG | This study |
| *dsbc* RT F | CGTCTTTACCGACGTGGAAT | This study |
| *dsbc* RT R | GGGTGTATTCCATCGACACC | This study |
| *rpoH* RT F | TGCACGAGTTCATCCTGAAG | This study |
| *rpoH* RT R | GCGACTCCATCTCCATCACT | This study |
| *hsp90xc* RT F | GACTGACCCAGCAGGAAATC | This study |
| *hsp90xc* RT R | AGATGTAGCAATGGCCCAAC | This study |
| *phoP* RT F | CGAAGAAGGCCTCTACATGG | This study |
| *phoP* RT R | CGGCTTGACCAAGTAATCGT | This study |
| *rpfB* RT F | CTTCGGCAAGACCATCACCT | This study |
| *rpfB* RT R | ACCAGATTGCGGTGAGTCAG | This study |
